# p140Cap Controls Female Fertility in Mice Acting *via* Glutamatergic Afference on Hypothalamic Gonadotropin-Releasing Hormone Neurons

**DOI:** 10.3389/fnins.2022.744693

**Published:** 2022-02-14

**Authors:** Mattia Camera, Isabella Russo, Valentina Zamboni, Alessandra Ammoni, Simona Rando, Alessandro Morellato, Irene Cimino, Costanza Angelini, Paolo Giacobini, Roberto Oleari, Federica Amoruso, Anna Cariboni, Isabelle Franceschini, Emilia Turco, Paola Defilippi, Giorgio R. Merlo

**Affiliations:** ^1^Department of Molecular Biotechnology and Health Sciences, University of Turin, Turin, Italy; ^2^Laboratory of Development and Plasticity of the Neuroendocrine Brain, Jean-Pierre Aubert Research Centre, Inserm U1172, Lille, France; ^3^Metabolic Research Laboratories, Wellcome Trust–Medical Research Council Institute of Metabolic Science, University of Cambridge, Cambridge, United Kingdom; ^4^Department of Pharmacological and Biomolecular Sciences, University of Milan, Milan, Italy; ^5^Physiologie de la Reproduction et des Comportements, French National Centre for Scientific Research, French Institute of the Horse and Riding, French National Research Institute for Agriculture, Food and Environment, Université de Tours, Nouzilly, France

**Keywords:** p140Cap, GnRH (Gonadotropin-Releasing Hormone), kisspeptin, glutamate, fertility

## Abstract

p140Cap, encoded by the gene *SRCIN1* (*SRC kinase signaling inhibitor 1)*, is an adaptor/scaffold protein highly expressed in the mouse brain, participating in several pre- and post-synaptic mechanisms. *p140Cap* knock-out (KO) female mice show severe hypofertility, delayed puberty onset, altered estrus cycle, reduced ovulation, and defective production of luteinizing hormone and estradiol during proestrus. We investigated the role of p140Cap in the development and maturation of the hypothalamic gonadotropic system. During embryonic development, migration of Gonadotropin-Releasing Hormone (GnRH) neurons from the nasal placode to the forebrain in *p140Cap* KO mice appeared normal, and young *p140Cap* KO animals showed a normal number of GnRH-immunoreactive (-ir) neurons. In contrast, adult *p140Cap* KO mice showed a significant loss of GnRH-ir neurons and a decreased density of GnRH-ir projections in the median eminence, accompanied by reduced levels of GnRH and LH mRNAs in the hypothalamus and pituitary gland, respectively. We examined the number of kisspeptin (KP) neurons in the rostral periventricular region of the third ventricle, the number of KP-ir fibers in the arcuate nucleus, and the number of KP-ir punctae on GnRH neurons but we found no significant changes. Consistently, the responsiveness to exogenous KP *in vivo* was unchanged, excluding a cell-autonomous defect on the GnRH neurons at the level of KP receptor or its signal transduction. Since glutamatergic signaling in the hypothalamus is critical for both puberty onset and modulation of GnRH secretion, we examined the density of glutamatergic synapses in *p140Cap* KO mice and observed a significant reduction in the density of VGLUT-ir punctae both in the preoptic area and on GnRH neurons. Our data suggest that the glutamatergic circuitry in the hypothalamus is altered in the absence of p140Cap and is required for female fertility.

## Introduction

The neuroendocrine control of sexual maturation and reproduction is a critical process, essential for the preservation of species. In mammals, the hypothalamic-pituitary-gonadal (HPG) axis exerts a tight regulation in all phases, controlling both puberty onset and fertility. Three major hierarchically organized anatomical components participate in the HPG axis: the hypothalamus, the pituitary gland, and the gonads. The HPG axis is centrally governed by the pulsatile release of Gonadotropin-Releasing Hormone (GnRH) into the pituitary portal system by the GnRH-secreting neurons whose cell bodies, in mice, are scattered within the medial septum (MS), preoptic area (POA), anterior hypothalamic area (AHA), and the *organum vasculosum* of the *lamina terminalis* (OVLT) (Jasoni et al., 2009).

During embryonic development, the first GnRH-immunoreactive (-ir) cells are first seen in the region of the olfactory placode and, although they have a mixed origin (Forni et al., 2011), they migrate along the axons of olfactory receptor neurons of the vomeronasal organ to reach the olfactory bulb primordium around E13, and then the septum-hypothalamic area (Cariboni et al., 2007). When GnRH neurons reach the hypothalamus, they engage in complex synaptic circuits with local and projection neurons and send their projections to the median eminence (ME), where they release GnRH into the pituitary portal bloodstream. Once released, GnRH acts on membrane-bound GnRH receptors present on endocrine cells of the anterior pituitary gland to induce the synthesis and secretion of Follicle-Stimulating Hormone (FSH) and Luteinizing Hormone (LH) (Grachev and Goodman, 2016).

In prepubertal mice, GnRH neurons show a complex morphology, with a highly branched dendritic tree, while in adult mice the vast majority of these neurons show 1 or 2 extensions from the soma, appearing unipolar or bipolar (Cottrell et al., 2006). However, the functional significance of this morphological maturation is yet to be determined.

Defects of GnRH neuron activity or migration are thought to be the primary cellular cause of congenital disorders known as Kallmann Syndrome and Central Hypogonadotropic Hypogonadism, characterized by delayed sexual maturation and hypofertility (Cariboni and Maggi, 2006; Boehm et al., 2015).

While migration and homing of GnRH neurons are well studied at the cellular and molecular level, other aspects remain poorly understood, such as the trophic factors that assure GnRH neuron survival until puberty and adult age, and the full array of neurotransmitters and neuropeptides that drive their final maturation and function (Clarkson and Herbison, 2006; Cottrell et al., 2006; Herbison, 2016).

In rodents, a wealth of evidence indicates that the secretory activity of GnRH neurons is modulated by neuropeptides, such as kisspeptin (KP) and neuropeptide Y (NPY), as well as classical neurotransmitters, such as glutamate, GABA, monoamines, and acetylcholine (Spergel, 2019a,b). Focusing on glutamate, it has been hypothesized that glutamatergic neurotransmission is critical for both the activation of GnRH neurons at the time of puberty and the modulation of GnRH secretion, required for fertility (Iremonger et al., 2010; Spergel, 2019a). For example, NMDA receptor agonists can induce precocious puberty, while NMDA receptor antagonists can delay puberty onset (Urbanski, 1990; Smyth and Wilkinson, 1994). Moreover, NMDA antagonists can inhibit either spontaneous or stimulated LH secretion in female rats (Urbanski, 1990; Brann and Mahesh, 1991). The effect of glutamate on GnRH neurons has been also investigated in several *in vitro* studies using immortalized GnRH-ir cell lines, which indicate a GnRH release-promoting activity of glutamate (Spergel et al., 1994; El-Etr et al., 2006).

The p140Cap protein (also known as SNIP), encoded by the gene *Srcin1*, is a docking/adaptor molecule that binds Src kinase and p130Cas (Stefano et al., 2004), coordinates the intracellular transduction of membrane receptors and cell adhesion signals, finely modulates cell responses, and has been implicated in integrin-dependent cell adhesion and signaling (Di Stefano et al., 2007). In the rodent brain, p140Cap is widely expressed in the cortex, cerebellum, hippocampus, and hypothalamus (Chin et al., 2000), and is localized at both the post-synaptic and the pre-synaptic compartments of excitatory synapses, where it is involved in the morphological and functional maturation of the synapse (Ito et al., 2008; Tomasoni et al., 2013; Li et al., 2017).

We noticed that *p140Cap* knock-out (KO) mice are severely hypofertile. Therefore, we decided to characterize the neuroendocrine gonadotropic system controlling sexual maturation and fertility in these mice. We reveal a non-cell-autonomous function of p140Cap for glutamatergic input on juvenile and adult GnRH neurons, which is required for efficient female fertility.

## Materials and Methods

### Mouse Strains and Fertility Assay

Mice with the targeted mutation of *p140Cap* have been previously described (Repetto et al., 2014). The mutation was maintained in homozygosity (as the animals are healthy, despite reduced fertility) in the transgenic unit of the Molecular Biotechnology Center (University of Turin, Turin, Italy) and animals were given water and food *ad libitum*. Genetic screening was performed by PCR as previously described (Repetto et al., 2014). Procedures were conducted in conformity with national and international laws and policies, protocols were approved by the internal Ethical Committee and authorized by the Italian Ministry of Health. To assess the fertility of both sexes, three possible mating combinations between *p140Cap* KO and WT P60 female and male mice were carried out. Measurements included the number of days required to produce the first litter, the numbers of litters born, and the number of pups per litter, recorded over 90 days.

### Puberty Onset and Estrous Cyclicity

Assessment of vaginal opening and examination of estrus cyclicity were carried out as previously described (Caligioni, 2009). Vaginal lavage of *p140Cap* KO females and their control littermates was performed every day (9:00 A.M. to 11:00 A.M.) for 20 consecutive days using 0.9% saline. Smears were observed under the microscope and the phase identified as diestrus/metestrus if they predominantly contained leukocytes, as proestrus if they predominantly contained nucleated cells, and as estrus if they predominantly contained cornified epithelial cells. An estrus cycle was considered normal when the vaginal lavage had leukocytes for 2 days followed by 1 day of nucleated cells and 1–2 days of cornified cells.

### Ovarian Histology and Quantitative Analysis

Ovaries were collected from P120 *p140Cap* KO and WT mice, fixed in 4% (w/v) PFA in PBS, and stored at 4°C. Paraffin-embedded ovaries were sectioned at a thickness of 5 μm and stained with hematoxylin-eosin. *Corpora lutea* and preovulatory follicles were counted on photomicrographs from every tenth section throughout the ovary.

### Hormonal Stimulation

Pregnant mare’s serum gonadotropin (5 IU/mouse) was inoculated intraperitoneally in P60 *p140Cap* KO and WT animals. 48 h later, human chorionic gonadotropin (5 IU/mouse) was administered intraperitoneally to stimulate ovulation. After 22 h, mice were sacrificed, the ovaries were removed, cells of the cumulus oophorus were eliminated by treatment with 0.3% hyaluronidase, and the oocytes were collected and counted. KP-54 (Tocris) was injected intraperitoneally (1 nmol in 100 μl of PBS/mouse) in P45 (for the determination of c-Fos-ir GnRH neurons) and P60 (for the quantification of serum LH levels) *p140Cap* KO and WT females in the diestrus phase of the estrous cycle. For the quantification of serum LH levels, animals were deeply anesthetized and blood was collected from the retro-orbital cavity 1 h after the injection. For the determination of c-Fos-ir GnRH neurons, animals were deeply anesthetized and transcardially perfused 90 min after the injection, and brains processed as described below.

### Brain Preparation and Histological Analysis

For the preparation of adult and P10 *p140Cap* KO and WT brains, mice were deeply anesthetized with Avertin (30 μl pure Avertin in 400 μl of PBS/mouse), transcardially perfused with 10 ml of PBS and then with 10 ml of 4% (w/v) PFA in PBS, and brains were dissected. For kisspeptin immunolabeling, P100 *p140Cap* KO and WT females in the proestrus phase of the estrous cycle were analyzed. For VGLUT-VGAT/GnRH double immunolabeling, P60 *p140Cap* KO and WT females in the diestrus phase of the estrous cycle were analyzed. For VGLUT-VGAT/KP double immunolabeling, P120 *p140Cap* KO and WT females in the proestrus phase of the estrous cycle were analyzed. For the preparation of embryonic brains, E14.5 embryos were collected by cesarean cut, and brains were dissected. After dissection, embryonic, P0, P10, and adult brains were post-fixed overnight at 4°C in 4% (w/v) PFA in PBS, placed overnight at 4°C in 30% (w/v) sucrose in PBS for cryoprotection, embedded in OCT blocks, and stored at –80°C until analysis. P0, P10, and adult brains were sliced into free-floating coronal sections of 30 μm using a cryotome (Leica CM1950). Free-floating sections were collected in PBS in multiwell plates and stored at –20°C in a cryoprotectant solution [30% (v/v) glycerol and 30% (v/v) ethylene glycol in 0.2 M phosphate buffer, pH 7.4] until processed for immunolabeling. Embryonic brains were sliced into sagittal sections of 16 μm using a cryotome, mounted on glass slides, and stored at –20°C until processed for immunolabeling.

### Immunohistochemistry

Immunohistochemistry to detect GnRH was performed as previously reported (Giacobini et al., 2008). For GnRH/p140Cap, GnRH/VGAT, GnRH/VGLUT, and c-Fos/GnRH double immunostainings, sections were blocked in 12% Normal Goat Serum and 2% Bovine Serum Albumin in 0.5% TritonX-100 in PBS for 1 h at room temperature (RT) and subsequently incubated with primary antibodies in 0.1% TritonX-100 in PBS for 48 h at 4°C. Sections were then washed in PBS, incubated with the secondary antibodies and DAPI (1:1000) diluted in 0.2% TritonX-100 in PBS for 2 h at RT, and washed three times in PBS. Staining for KP/GnRH was carried out as described elsewhere (Naulé et al., 2014). For KP/VGLUT and KP/VGAT double immunostainings, sections were blocked in 2% Normal Goat Serum and 0.2% Bovine Serum Albumin in 0.2% TritonX-100 in PBS for 1 h at RT. Sections were then incubated with anti-KP primary antibody in 0.2% TritonX-100 in PBS for 48 h at 4°C, incubated with anti-sheep secondary antibody diluted in 0.2% TritonX-100 in PBS for 2 h at RT, incubated with anti-VGLUT/VGAT primary antibody in 0.2% TritonX-100 in PBS for 48 h at 4°C, incubated with anti-guinea pig secondary antibody diluted in 0.2% TritonX-100 in PBS for 2 h at RT, and finally washed three times in PBS. GnRH/activated caspase 3 immunostainings were carried out as described elsewhere (Macchi et al., 2017). Sections were mounted on glass slides using 1,4-diazabicyclo [2.2.2] octane (Sigma-Aldrich) and examined. In all steps, *p140Cap* KO and WT sections were processed in parallel at the same time. The total number of GnRH-ir neurons in the adult brain was determined as described elsewhere (Herbison et al., 2008). Determination of the total number of GnRH-ir neurons in the embryonic brain was carried out by sectioning whole brains in sagittal orientation at 16 μm. GnRH-ir neurons were counted in every third section through the brain, and the number obtained was multiplied by 3 to get the total number of GnRH neurons. Quantification of GnRH immunoreactivity (voxel counts) was carried out on anatomically matched sections of the median eminence [plates 47-50 of the Mouse Brain Atlas of Paxinos et Franklin (Paxinos and Franklin, 2001)]. Determination of the number of kisspeptin neurons was carried out in anatomically matched sections of the anteroventral periventricular region (plates 28–29) and the rostral (plate 30) and caudal (plates 31–32) regions of the RP3V. Quantification of kisspeptin immunoreactivity (voxel counts) was carried out on anatomically matched sections of the arcuate nucleus (plate 41-45). Determination of the number of VGLUT-ir *punctae* in the OVLT and POA was carried out on anatomically matched sections (plates 25-27).

### *In situ* Hybridization

*In situ* hybridization was carried out as previously described (Oleari et al., 2021). Briefly, PFA-fixed 20 μm thick cryosections were incubated with digoxigenin (DIG)-labeled anti-sense riboprobes for mouse Gnrh1 at 65°C. mRNA expression was revealed with AP-conjugated anti-DIG antibody (1:1500; Roche), 4-Nitro blue tetrazolium chloride solution, and 5-Bromo-4-chloro-3-indolyl phosphate disodium salt (1:1000, Roche).

### Antibodies

Primary antibodies used are: rabbit anti-GnRH (Beauvillain and Tramu, 1980), sheep anti-KP AC053 (Franceschini et al., 2013), sheep anti-GnRH (Skrapits et al., 2015), mouse anti-p140Cap (Grasso et al., 2018, see Supplementary Figure 1 for specificity test), guinea pig anti-Vesicular GABA Transporter (VGAT, Synaptic System, used 1:500), guinea pig anti-Vesicular glutamate Transporter (VGLUT, Synaptic System, used 1:500), rabbit anti-c-Fos (sc-52 Santacruz, used 1:500) and rabbit anti-cleaved caspase 3 (Cell Signaling Technology, used 1:400, Whittaker et al., 2021). Secondary antibodies used are: Alexa-Fluor 488 goat anti-rabbit (Invitrogen, used 1:400), Cy3 goat anti-rabbit (Invitrogen, used 1:800), Alexa-Fluor 568 goat anti-mouse (Invitrogen, used 1:400), FITC anti-guinea pig (Sigma F6261, used 1:100), Cy3 donkey anti-sheep (Jackson, used 1:1000). Peroxidase-conjugated secondary antibodies were obtained from GE Healthcare.

### Blood Sampling and Hormonal Assays

Following cycle stage determination by vaginal smear microscopic analysis, mice were anesthetized in the afternoon of proestrus and at the diestrus stage, and blood was collected from the retro-orbital cavity. Serum LH was measured using Rodent LH ELISA kit (ERKR7017, Endocrine Technologies). Serum E2 was measured using Rodent estradiol ELISA kit (ERKR7011, Endocrine Technologies).

### Photo-Documentation and Image Analysis

Images were captured using a Nikon microscope (Eclipse 80i) and 2 × /0.06 NA, 10 × /0.30 NA, and 20 × /0.50 NA objectives (Nikon) equipped with a digital camera (CX 9000; MBF Bioscience). For observation coupled with confocal analysis, a laser-scanning Fluoview confocal system (IX70; Olympus) and 10 × /0.30 NA, 20 × /0.70 NA, and 60 × /1.25 NA objectives (Olympus) were used. Subsequent analysis of digitized images was performed with ImageJ (NIH, Bethesda, Maryland^1^) software. For the determination of c-Fos-ir GnRH neurons, c-Fos-ir/GnRH-ir double-stained neurons in the hypothalamus were counted and expressed as a percentage of the total number of GnRH-ir neurons. Quantification of kisspeptin and GnRH-ir fiber density was carried out by voxel counts on a set of 10 serial image planes (z step size = 1 μm). KP-ir, VGLUT-ir, and VGAT-ir *punctae* on GnRH neurons were counted manually in a set of 20 serial image planes (z step size = 0.5 μm). Only GnRH neurons whose cell bodies were entirely present in the slice depth were considered for the analysis. The density of VGLUT-ir *punctae* in the OVLT and POA was calculated using the function “Find Maxima” of ImageJ. Photoshop (Adobe) software was used to process, adjust and merge the photomontages.

### Real-Time Quantitative PCR for Gonadotropin-Releasing Hormone and Luteinizing Hormone mRNA Levels

Total RNA was extracted from dessected hypothalami and pituitary glands from *p140Cap* KO and WT mice using Trizol Reagent (Ambion, Life Technologies Italia) and its concentration was determined with a NanoDrop™ 1100 (NanoDrop Technologies, Wilmington, DE, United States). Total RNA was reverse transcribed with high-capacity cDNA reverse transcriptase (#4368813, Applied BioSystem) according to the manufacturer’s instructions and amplified with specific primers. Taqman PCR reactions were performed using the Universal Probe Library system (Roche Italia, Monza, Italy) and quantified with the Molecular Analyst software (Bio-Rad Laboratories). The 18S rRNA pre-developed TaqMan assay (#4319413, Applied Biosystems) was used as an internal control. The expression of the target genes was calibrated against the values obtained in WT animals. Primers and probes used:

GnRH f 5′- CCCTTTGACTTTCACATCCAA-3′

GnRH r 5′- CGCAACCCATAGGACCAGT-3′ [probe #19]

LH f 5′- GTCCCAGGACTCAACCAATG-3′

LH r 5′- AACACCTGCTGGTGGTGAA-3′ [probe #10].

### Statistical Analysis

For the statistical comparison, GraphPad Prism software (GraphPad Software Inc.) was used. For each experiment, the statistical test used is reported in the figure legends. Shapiro-Wilk and Kolmogorov-Smirnov tests were used to test for normality, F test was used to test for equality of variance, and results were evaluated to choose the appropriate statistical test.

## Results

### *p140Cap* KO Females Exhibit Reduced Fertility

**FIGURE 1:**
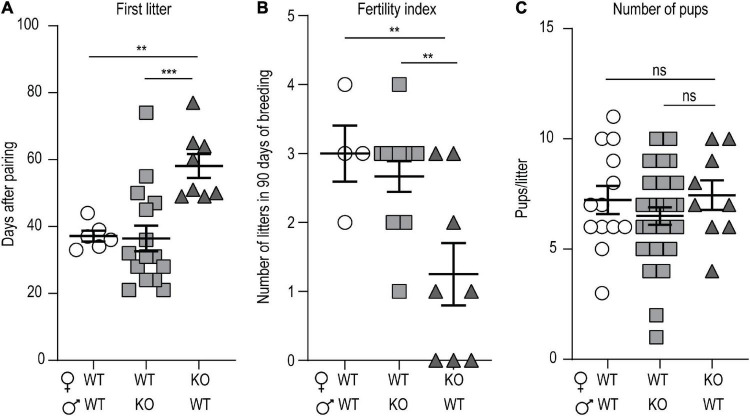
*p140Cap* KO female mice are hypofertile. **(A)** Number of days required to produce the first litter. *n* = 7 matings WT × WT; 15 matings WT female × *p140Cap* KO male; 8 matings *140Cap* KO female × WT male. Kruskal-Wallis test, *P* = 0.002; *post hoc* Dunn’s test, *P*_(WT__×__*WT)*_ = 0.02, *P*_(WT female__×_ *_*p*140*Cap*_*_*KO male)*_ < 0.001. **(B)** Total number of litters per mating over 90 days. *n* = 4 matings WT × WT; 12 matings WT female × *p140Cap* KO male; 8 matings *140Cap* KO female × WT males. Kruskal-Wallis test, *P* = 0.03; *post hoc* Dunn’s test, *P*_(WT__×__*WT)*_ = 0.02, *P*_(WT female__×_ *_*p*140*Cap*_*_*KO male)*_ = 0.02. **(C)** Number of pups per litter. *n* = 12 litters WT × WT; 24 litters WT female × *p140Cap* KO male; 9 litters *140Cap* KO female × WT males. Kruskal-Wallis test, *P* = 0.44. Data are represented as means ± SEM. ** = *P* < 0.01, *** = *P* < 0.001, ns = not significant.

We noticed that *p140Cap* KO mice have a lower mating success as compared to WT, even if they do not show any apparent behavioral deficit that could be accounted for hypofertility. To quantify the hypofertility and to assess whether both male and female fertility was affected, we set up a continuous mating protocol of 90 days, comparing different breeding combinations of P60 mice. In matings involving WT females and KO males, the number of days required to produce the first litter (Figure 1A), the fertility index (Figure 1B), determined as the number of litters obtained in 90 days, and the number of pups/litter (Figure 1C), were similar to matings between WT animals. In contrast, in matings involving KO females and WT males, both the number of days required to produce the first litter (Figure 1A) and the fertility index (Figure 1B) were significantly lower, while the number of pups/litter was normal (Figure 1C). These results suggest that the loss of p140Cap significantly alters female, but not male, fertility.

### *p140Cap* KO Females Show Impaired Ovulation and Altered Hormonal Status

**FIGURE 2:**
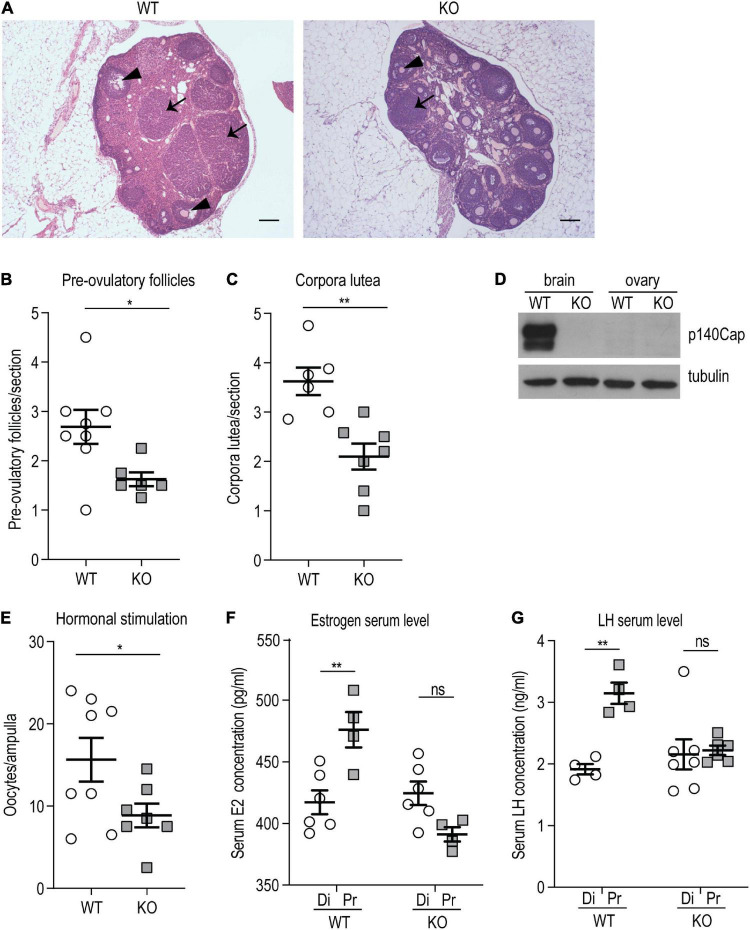
*p140Cap* KO female mice show ovarian defects. **(A)** Representative images of ovary sections from P120 *p140Cap* KO and WT mice. Arrows indicate *corpora lutea*, arrowheads indicate pre-ovulatory follicles. Scale bar = 500 μm. **(B)** Number of pre-ovulatory follicles per section in *p140Cap* KO and WT ovaries. *n* = 8 ovaries WT; 6 ovaries KO. Unpaired, two-tailed Welch’s *t*-test, *P* = 0.02. **(C)** Number of corpora lutea per section in *p140Cap* KO and WT ovaries. *n* = 6 ovaries WT; 7 ovaries KO. Unpaired, two-tailed *t*-test, *P* = 0.002. **(D)** Protein level of p140Cap expression in the total brain and ovary of *p140Cap* KO and WT mice, evaluated by western blot. Protein extracts from *p140Cap* KO and WT tissues were run in 6% SDS-PAGE. Membranes were decorated with anti-p140Cap antibody (top) and anti-tubulin antibody, as loading control (bottom). **(E)** Number of oocytes per ampulla from *p140Cap* KO and WT mice. Ovulation was stimulated by the injection of PMSG and HCG. *n* = 8 mice WT; 7 mice KO. Unpaired, two-tailed *t*-test, P = 0.05. **(F)** Estrogen (E2) serum levels in *p140Cap* KO and WT female mice measured by ELISA at diestrus and proestrus. *n* = 6 and 4 WT mice at diestrus and proestrus, respectively; 6 and 4 *p140Cap* KO mice at diestrus and proestrus, respectively. One-way ANOVA, *P* = 0.002; Sidak’s multiple comparison test, *P*_(proestrus WT vs_ _*diestrus*_ _WT)_ = 0.002; *P*_(proestrus KO vs_ _*diestrus*_ _KO)_ = 0.96. **(G)** LH serum levels in *p140Cap* KO and WT female mice measured by ELISA at diestrus and proestrus. *n* = 4 WT mice at diestrus and proestrus; 7 *p140Cap* KO mice at diestrus and 6 at proestrus. One-way ANOVA, *P* < 0.001; Sidak’s multiple comparison test, *P*_(proestrus WT vs_ _*diestrus*_ _WT)_ = 0.002; *P*_(proestrus KO vs_ _*diestrus*_ _KO)_ = 0.08. Di = diestrus; Pr = proestrus. Data are represented as means ± SEM. * = *P* < 0.05, ** = *P* < 0.01, ns = not significant.

Considering that the lack of p140Cap impacted only the reproductive axis in females and not in males, female mice were further characterized. To determine whether hypofertility was associated with ovulation deficiency, we evaluated ovarian morphology and follicle classification in P120 *p140Cap* KO and WT mice. Histological examination of *p140Cap* KO ovaries revealed a significant reduction in the number of pre-ovulatory follicles and *corpora lutea* as compared to WT (Figures 2A–C).

The defect observed in the ovarian compartment is apparently in contrast with the unaltered litter size observed in *p140Cap* KO mice. However, considering that several parameters can affect the number of pups at each pregnancy, such as efficiency of implantation, placenta functionality, and frequency of miscarriage, the reduced ovulation efficiency may be compensated, resulting in normal litter size. The impaired ovulation may depend on the loss of p140Cap expression in the ovarian tissue. Therefore, p140Cap expression was assessed by Western blot analysis on tissue extracts from the ovary and total brain of *p140Cap* KO and WT mice. As shown in Figure 2D, while p140Cap is highly expressed in the brain, it is undetectable in ovary extracts, indicating that the ovarian phenotype is non-cell-autonomous and may depend on the upstream hormonal axis.

To test if the reduced ovulation in *p140Cap* KO females was due to an impaired hormonal stimulation, a classical protocol of exogenous stimulation with Pregnan’ Mare’s Serum Gonadotropin (PMSG) and Human Chorionic Gonadotropin (HCG) was performed. As shown in Figure 2E, a relevant number of oocytes was found in both *p140Cap* KO and WT mice, indicating that in *p140Cap* KO females the ovarian tissue is still able to respond to an exogenous hormonal stimulation. However, while super-ovulation led to a mean of 20 oocytes/ampulla in WT mice, *p140Cap* KO females produced < 10 oocytes/ampulla. These results indicate that, although the ovarian tissue of KO mice may be less responsive, it is still able to undergo effective ovulation upon proper stimulation.

Ovulation of mature follicles in the ovary is induced by a large burst of GnRH-induced LH secretion—the preovulatory LH surge—which is triggered by increased estradiol (E2) levels. To assess the hormonal status in *p140Cap* KO mice, serum E2 and LH were quantified by ELISA in diestrus and proestrus phases. The results show that E2 and LH serum levels in *p140Cap* KO females do not increase during proestrus (Figures 2F,G), meaning that *p140Cap* KO females do not show the expected E2 and LH surge during proestrus, implying that these mice have a defective LH and E2 production in the proestrus stage.

### *p140Cap* KO Females Display Delayed Puberty Onset and Abnormal Estrous Cyclicity

**FIGURE 3:**
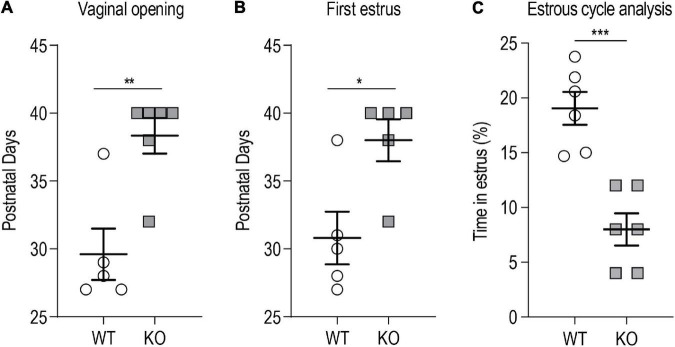
*p140Cap* KO female mice show delayed puberty and abnormal estrous cyclicity. **(A)** Postnatal day at which the vaginal opening appears. *n* = 5 WT mice; 6 *p140Cap* KO mice. Mann-Whitney test, *P* = 0.009. **(B)** Postnatal day at which the first estrus (defined by the presence of a majority of cornified epithelial cells in vaginal smears) occurred. *n* = 5 WT mice; 5 *p140Cap* KO mice. Mann-Whitney test, *P* = 0.02. **(C)** Percentage of time spent in estrus phase during 20 days of observation. *n* = 6 WT mice; 6 *p140Cap* KO mice. Unpaired, two-tailed *t*-test, *P* < 0.001. Data are represented as means ± SEM. * = *P* < 0.05, ** = *P* < 0.01, *** = *P* < 0.001, ns = not significant.

We evaluated vaginal opening as an external index of puberty onset. Vaginal opening in female rodents is dependent on E2 levels and it reflects the maturation of the mouse female genital tract at the time of puberty, dependent on the activation of the HPG axis (Kennedy and Armstrong, 1973; Rodriguez et al., 1997). Weaned *p140Cap* KO and WT P21 females were daily examined for vaginal opening and the appearance of the first estrus, by flushing of vaginal secretion. Both vaginal opening and first estrus were significantly delayed in *p140Cap* KO females (Figures 3A,B). We also analyzed estrous cyclicity of *p140Cap* KO female mice by daily inspection of vaginal cytology. We found that *p140Cap* KO animals spend only 5% of time in the estrus phase, while WT females spend more than 20% of time (Figure 3C and Supplementary Figure 2), indicating that *p140Cap* KO mice have altered estrous cyclicity.

### *p140Cap* Is Detected in Embryonic, but Not in Adult, Gonadotropin-Releasing Hormone Neurons

**FIGURE 4:**
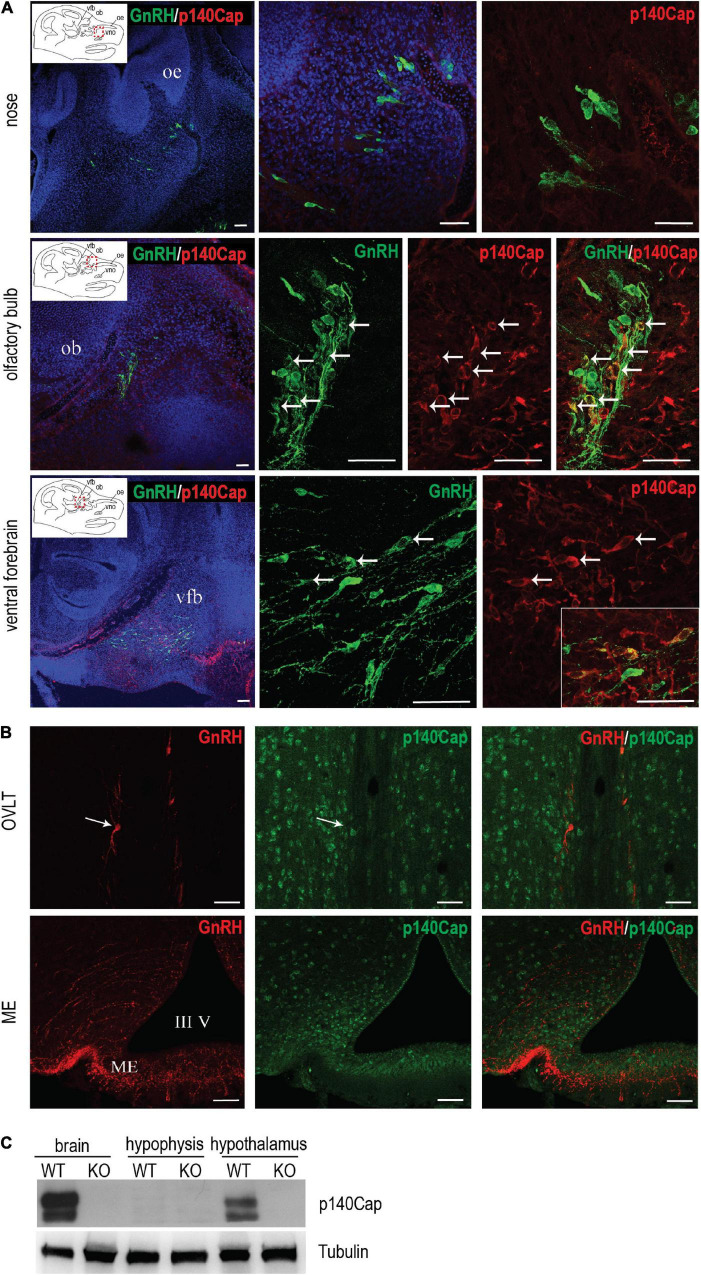
p140Cap is expressed in a subpopulation of GnRH neurons during embryonic development. **(A)** Confocal optical sections of the expression of GnRH (green) and p140Cap (red) in the nose, olfactory bulb, and ventral forebrain of E14.5 WT embryos. Sections were counterstained with DAPI (blue). oe = olfactory epithelium; ob = olfactory bulb; vfb = ventral forebrain, vno = vomeronasal organ. Scale bars: 50 μm. **(B)** Confocal optical sections of the expression of GnRH (red) and p140Cap (green) in the *organum vasculosum* of the *laminae terminalis* (OVLT) and median eminence (ME) of WT P60 mice. Sections were counterstained with DAPI (blue). III V = 3^rd^ ventricle. Scale bar: 50 μm. **(C)** Protein level of p140Cap in the total brain, hypophysis, and hypothalamus of *p140Cap* KO and WT P60 mice, evaluated by western blot. Protein extracts were run in 6% SDS-PAGE. Membranes were decorated with anti-p140Cap antibody (top) and anti-tubulin antibody, as a loading control (bottom).

The endocrine and ovarian phenotypes described above clearly point to a central defect of GnRH neurons. Thus, we set forth to examine the expression and localization of p140Cap in the nasal-olfactory region of the developing embryo, and in the adult hypothalamus, by double immunostaining with anti-p140Cap and anti-GnRH antibodies, followed by confocal microscopy. At earlier embryonic ages, co-expression was clearly detected in a subpopulation (about 25–30%) of GnRH-ir neurons in the olfactory region and ventral forebrain (Figure 4A). When we analyzed sections of the adult hypothalamus, p140Cap was not detected in GnRH-ir neurons (Figure 4B), although it was detected in the vast majority of cells in this region.

Finally, we examined the expression of p140Cap on tissue extracts from the pituitary gland and hypothalamus by western blot analysis. While p140Cap is undetectable in pituitary gland extracts, it is highly expressed in the hypothalamus (Figure 4C), consistently with the immunostaining results.

### *p140Cap* KO Females Show Reduced Gonadotropin-Releasing Hormone mRNA Level, Reduced Number of Gonadotropin-Releasing Hormone Neurons, and Reduced Gonadotropin-Releasing Hormone Innervation at the Median Eminence

**FIGURE 5:**
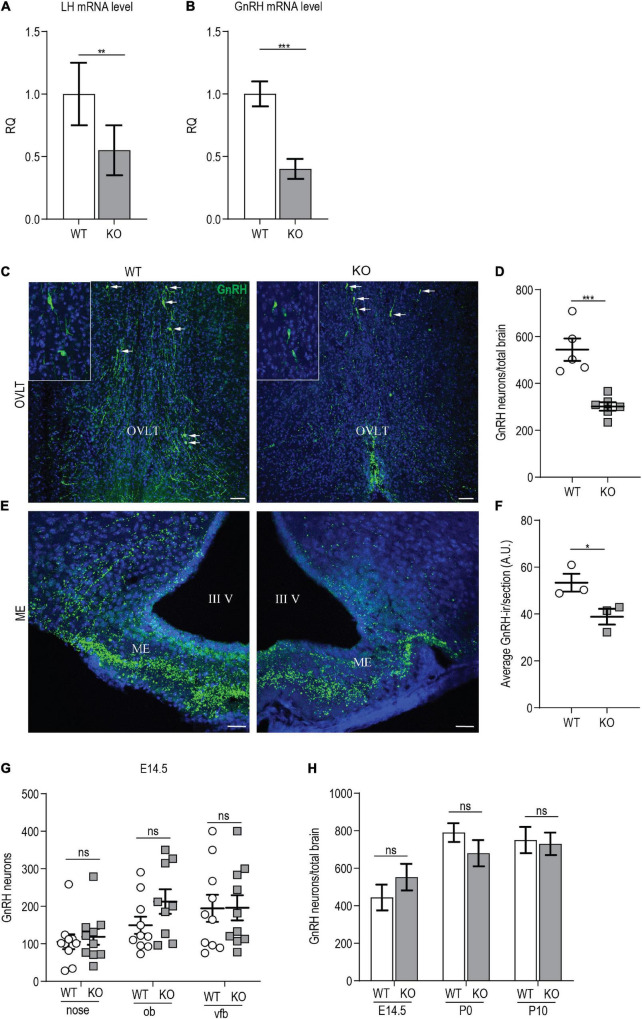
*p140Cap* KO females show reduced number of GnRH neurons. **(A)** LH mRNA expression in the hypophysis of *p140Cap* KO and WT adult females, evaluated by real-time quantitative PCR. *n* = 12 WT mice; 15 KO mice. Unpaired, two-tailed *t*-test, *P* = 0.006. **(B)** GnRH mRNA expression in the hypothalamus of *p140Cap* KO and WT adult females, evaluated by real-time quantitative PCR. *n* = 4 WT mice; 6 KO mice. Unpaired, two-tailed *t*-test, *P* < 0.001. **(C)** Maximum intensity projections of z-stack images (10 serial image planes; z step size = 1 μm) of the expression of GnRH (green) in the OVLT and ME of adult *p140Cap* KO and WT adult female brains. Sections were counterstained with DAPI (blue). Scale bar: 75 μm. **(D)** Number of GnRH neurons in the hypothalamus of adult *p140Cap* KO and WT females. *n* = 5 WT mice; 6 *p140Cap* KO mice. Unpaired, two-tailed *t*-test, *P* < 0.001. **(E)** Maximum intensity projections of z-stack images (10 serial image planes; z step size = 1 μm) of the expression of GnRH (green) in the median eminence of adult *p140Cap* KO and WT mice. Sections were counterstained with DAPI (blue). ME, median eminence; III V, 3*^rd^* ventricle. Scale bar: 75 μm. **(F)** Average GnRH-immunoreactivity (-ir) in the median eminence of adult *p140Cap* KO and WT females. *n* = 3 WT mice; 3 *p140Cap* KO mice; at least 5 sections were analyzed for each mouse. Unpaired, two-tailed *t*-test, *P* = 0.05. **(G)** Number of GnRH neurons in various regions of E14.5 *p140Cap* KO and WT embryos. ob, olfactory bulb; vfb, ventral forebrain. *n* = 10 WT brains; 10 *p140Cap* KO brains. Two-way ANOVA, *P*_(WT vs KO)_ = 0.27. **(H)** Number of GnRH neurons in the hypothalamus of E14.5, P0, and P10 *p140Cap* KO and WT mice. *n* = 10 E14.5, 5 P0 and 5 P10 WT mice; 10 E14.5, 6 P0 and 6 P10 *p140Cap* KO mice. Two-way ANOVA, *P*_(WT vs KO)_ = 0.44. Data are represented as means ± SEM. * = *P* < 0.05, ** = *P* < 0.01, *** = *P* < 0.001, ns = not significant.

To define which component of the HPG axis is affected by the absence of p140Cap, we analyzed the amount of GnRH and LH transcripts in the hypothalamus and pituitary gland of *p140Cap* KO females. Real-time quantitative PCR analysis showed a marked decrease in both LH expression in the pituitary (Figure 5A) and GnRH expression in the hypothalamus (Figure 5B) of *p140Cap* KO females, as compared to WT. These data indicate that, in the absence of *p140Cap*, the hypothalamus is defective in the expression of GnRH mRNA, with a consequent negative impact on LH expression. These results also imply that the defect in *p140Cap* KO mice may depend on impaired GnRH neuronal function.

Next, we evaluated the number of GnRH neurons in the MS, POA, AHA, and OVLT of *p140Cap* KO and WT P60 females, and we detected a significant 40% reduction in the total number of GnRH neurons in the mutant mice (Figures 5C,D), which is consistent with the reduced levels of GnRH mRNA. No ectopic localization of GnRH neurons was observed in mutant mice.

To verify that the loss of GnRH-ir neurons resulted from the lack of GnRH mRNA, and not from defects in GnRH prohormone processing, we conducted *in situ* hybridization. We found a significant reduction in the number of GnRH mRNA-containing neurons in *p140Cap* KO adult females as compared to WT (Supplementary Figures 3A,B).

GnRH neurons project their axons to the ME, where GnRH is released into the pituitary portal blood for delivery to the anterior pituitary, eliciting the secretion of LH and FSH. To assess if the reduction in the number of GnRH neurons resulted in a reduced number of GnRH innervation in the ME, we analyzed the ME of P60 females, by immunostaining with anti-GnRH. The results show a reduction of GnRH-immunoreactivity in *p140Cap* KO mice as compared with WT (Figures 5E,F).

A reduced number of hypothalamic GnRH neurons may result from an altered migration during embryonic development, impaired neurogenesis, or altered survival of GnRH neurons. To determine whether the absence of the p140Cap may impact the migration of GnRH embryonic neurons, we analyzed the nasal-olfactory region, the olfactory bulbs, and the ventral forebrain of *p140Cap* KO and WT E14.5 embryos. As shown in Figure 5G, there was no significant variation in the number of GnRH neurons between *p140Cap* KO and WT embryos in the various regions, indicating that the absence of p140Cap does not cause migratory defects of immature GnRH neurons during embryonic development. We also found no difference in the total number of GnRH neurons present in the hypothalamus of *p140Cap* KO and WT mice at E14.5, P0, and P10 (Figure 5H), indicating that the lower number of GnRH neurons in adult *p140Cap* KO mice does not result from impaired neurogenesis. Finally, to assess if the decreased number of GnRH neurons was due to increased cell death, we labeled adjacent coronal sections of the medial POA (MPOA, where most GnRH neurons reside) in P30 females, with antibodies against GnRH or activated caspase-3, a marker for cells committed to undergo apoptosis. Only a few apoptotic cells were detected in the MPOA of *p140Cap* KO and WT mice (1-2 cells per animal, Supplementary Figure 3C), suggesting that the loss of GnRH neurons in *p140Cap* KO mice is not due to increased apoptosis. Therefore, we were not able to determine the mechanism underlying the loss of GnRH neurons in *p140Cap* KO mice.

### *p140Cap* KO Females Show Delayed Maturation of Gonadotropin-Releasing Hormone Neurons

Having excluded embryonic migratory defects of GnRH neurons, a reduced number of hypothalamic GnRH neurons may result from post-migratory early-postnatal defects. We examined the morphological maturation of GnRH neurons in the hypothalamus of *p140Cap* KO and WT mice. We determined the fraction of GnRH neurons showing a unipolar/bipolar or a multipolar morphology in the brains of *p140Cap* KO and WT P10 female mice. In *p140Cap* KO brains, 34% of GnRH neurons were multipolar and 66% were bipolar, while in WT brains the fractions were 20% and 80%, respectively (Supplementary Figure 4A). The same analysis was conducted on brains from P60 (young adults) females, but we observed no change in the morphology of GnRH neurons (Supplementary Figure 4B). These results indicate that, in the absence of p140Cap, juvenile hypothalamic GnRH neurons are delayed in their morphological maturation.

### *p140Cap* KO Females Show a Normal Kisspeptin Neuroanatomy and a Normal Gonadotropin-Releasing Hormone/Luteinizing Hormone Response to Exogenous Kisspeptin

**FIGURE 6:**
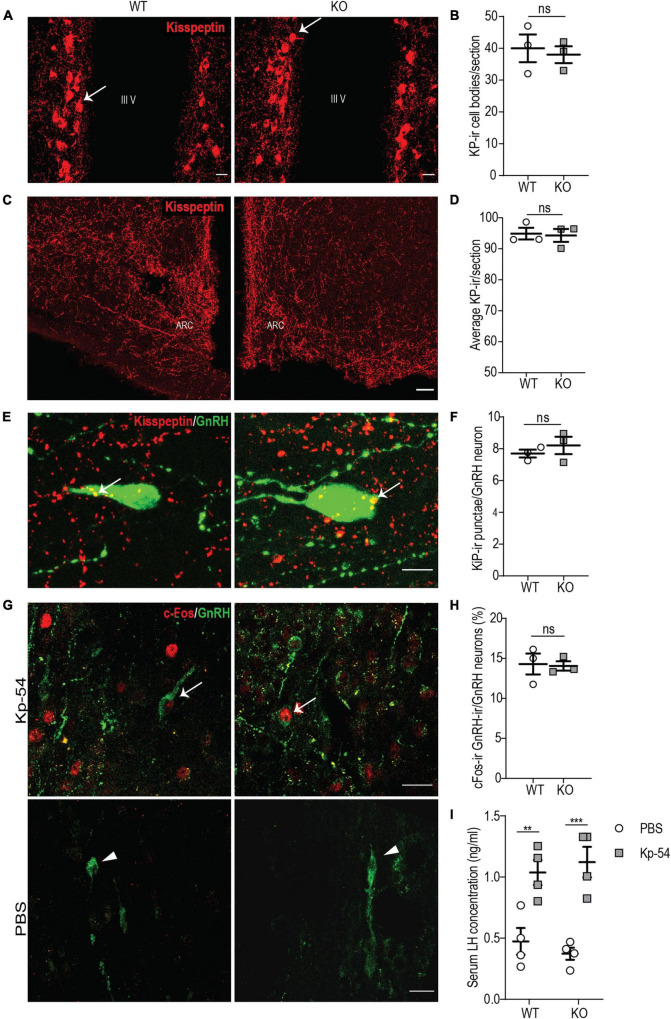
*p140Cap* KO females show a normal KP neuroanatomy and a normal GnRH/LH response to exogenous KP. **(A)** Maximum intensity projections of z-stack images (5 serial image planes; z step size = 2 μm) of KP neurons in the RP3V region of the hypothalamus of P60 *p140Cap* KO and WT female mice. Arrows indicate KP neurons; III V, 3^rd^ ventricle. Scale bar: 10 μm. **(B)** Number of KP neurons in the RP3V of *p140Cap* KO and WT P60 female mice. *n* = 3 WT mice; 3 *p140Cap* KO mice; at least 4 sections were analyzed for each mouse. Unpaired, two-tailed *t*-test, *P* = 0.71. **(C)** Maximum intensity projections of z-stack images (10 serial image planes; z step size = 1 μm) of KP fibers in the arcuate nucleus of P60 *p140Cap* KO and WT mice. ARC, arcuate nucleus of the hypothalamus. Scale bar: 50 μm. **(D)** Average KP immunoreactivity (-ir) in the arcuate nucleus of P60 *p140Cap* KO and WT mice. *n* = 3 WT mice; 3 *p140Cap* KO mice; at least 5 sections were analyzed for each mouse. Mann-Whitney test, *P* > 0.99 **(E)** Maximum intensity projections of z-stack images (20 serial image planes; z step size = 0.5 μm) of KP-ir *punctae* (red) and GnRH neurons (green) in P60 *p140Cap* KO and WT mice. Arrows indicate KP-ir *punctae* on GnRH neurons. Scale bar: 10 μm. **(F)** Average number of KP-ir *punctae* on GnRH neurons in P60 *p140Cap* KO and WT mice. *n* = 3 WT mice; 3 *p140Cap* KO mice; at least 30 GnRH neurons were analyzed for each mouse. Unpaired, two-tailed *t*-test, *P* = 0.45. **(G)** Maximum intensity projections of z-stack images (10 serial image planes; z step size = 1 μm) of the expression of c-Fos (red) and GnRH (green) in *p140Cap* KO and WT female mice treated with 1 nmol of Kp-54 or PBS, as control. Arrows indicate double-positive c-Fos-ir/GnRH-ir neurons. Scale bar: 20 μm. **(H)** Number of double-positive c-Fos-ir/GnRH-ir neurons in *p140Cap* KO and WT female treated with 1 nmol of Kp-54 or PBS, as control. *n* = 3 WT mice; 3 p140Cap KO mice; at least 50 neurons were analyzed for each mouse. Unpaired, two-tailed *t*-test, *P* = 0.88. **(I)** Serum LH levels in *p140Cap* KO and WT female mice treated with 1 nmol of Kp-54 or PBS, as control. *n* = 3 WT mice; 3 *p140Cap* KO mice. Two-way ANOVA, *P*_(WT vs KO)_ = 0.94, *P*_(PBS vs Kp–54)_ < 0.001, *post hoc* Tukey’s test, *P*_(*WT)*_ = 0.009. *P*_(KO)_ < 0.001. Data are represented as means ± SEM. ** = *P* < 0.01, *** = *P* < 0.001, ns = not significant.

Since the sole reduction in the number of GnRH neurons is unlikely to account for the hypofertility observed in *p140Cap* KO mice (Herbison et al., 2008), we further analyzed the KP system. The KP system, consisting of two groups of KP neurons located in the arcuate nucleus (ARC) and in the rostral periventricular region of the third ventricle (RP3V), has a crucial role in the control of GnRH neurons activity and pulsatility (Clarkson et al., 2009; Harter et al., 2018). Considering the wide distribution of p140Cap expression in the mouse brain, we hypothesized that a defect in the endogenous KP neuroendocrine system could affect the function/activity of GnRH neurons. Thus, we set forth to determine whether alteration of the KP system could account for the reduced function of GnRH neurons in *p140Cap* KO females. We double-immunostained anatomically matched hypothalamic sections of *p140Cap* KO and WT female brains for KP and GnRH and determined the number of KP neurons in the RP3V, the density of KP-ir fibers in the ARC, and the number of KP-ir *punctae* in close apposition to GnRH neurons. In the absence of p140Cap, we observed no changes in the number of KP neurons in the RP3V (Figures 6A,B), in the density of KP-ir fibers in the ARC (Figures 6C,D), and in the number of KP-ir *punctae* in close apposition to GnRH neurons (Figures 6E,F).

Next, we decided to further probe the immediate responsiveness of GnRH neurons to exogenous KP, as a way to monitor the presence of a normal KP receptor/transduction machinery. To do this, we used an experimental paradigm previously adopted by other authors (Chen et al., 2014; León et al., 2016): we treated P45 *p140Cap* KO and WT females in the diestrus phase of the estrus cycle with 1 nmol of KP-54 and, after 1 h, we collected the hypothalamus, on which we determined the fraction of GnRH neurons that were immunoreactive for c-Fos in their nuclei, by immunostaining. The results show that, upon treatment with KP-54, *p140Cap* KO and WT mice had a similar fraction of GnRH-ir/c-Fos-ir double-positive cells (Figures 6G,H), suggesting unaltered responsiveness.

To further sustain this result, we probed for the surge of LH in the blood of *p140Cap* KO and WT animals, collected 1 h after treatment with KP-54. Also in this case, we did not observe changes in the LH surge in *p140Cap* KO versus the WT samples (Figure 6I). Together, these results indicate that, in the absence of p140Cap, hypothalamic GnRH neurons show a normal response to exogenous KP, excluding a cell autonomous defect of GnRH neurons at the level of KP receptor or its signal transduction.

### *p140Cap* KO Females Show a Reduced Number of Glutamatergic Synapses in the *Organum Vasculosum* of the *Lamina Terminalis* and on Gonadotropin-Releasing Hormone Neurons

Two other key elements controlling the activity of GnRH neurons are the glutamatergic and the GABAergic synaptic stimulation on these neurons. Studies on immortalized cell lines (such as GT1-7 cells) and on the isolated hypothalamus showed that glutamatergic stimulation positively regulates GnRH release (Spergel et al., 1994; El-Etr et al., 2006). Since p140Cap is expressed in both the pre- and the post-synaptic compartments of glutamatergic synapses and has been shown to be essential for effective glutamatergic synaptogenesis and maturation of dendritic spines (Jaworski et al., 2009; Tomasoni et al., 2013; Repetto et al., 2014), we hypothesized that defects in glutamatergic synapses may contribute to the hypofertility of *p140Cap* KO female mice.

**FIGURE 7:**
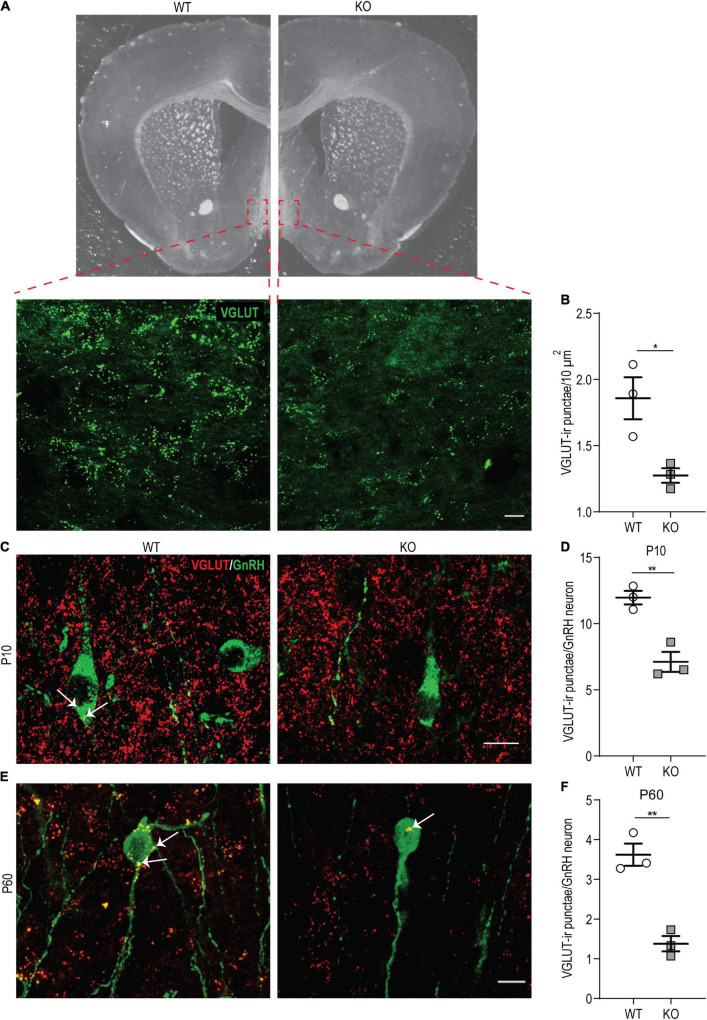
*p140Cap* KO females show reduced density of VGLUT-ir punctae in the hypothalamus. **(A)** Maximum intensity projections of z-stack images (20 serial image planes; z step size = 0.5 μm) of VGLUT-ir *punctae* in the OVLT of *p140Cap* KO and WT mice. Scale bar: 20 μm. **(B)** Average number of VGLUT-ir *punctae* per 10 μm^2^ in the OVLT and POA of *p140Cap* KO and WT mice. *n* = 3 WT mice; 3 *p140Cap* KO mice; at least 10 sections were analyzed for each mouse. Unpaired, two-tailed *t*-test, *P* = 0.03. **(C)** Maximum intensity projections of z-stack images (20 serial image planes; z step size = 0.5 μm) of VGLUT-ir *punctae* (red) and GnRH neurons (green) in P10 *p140Cap* KO and WT mice. Arrows indicate VGLUT-ir *punctae* on GnRH neurons. Scale bar: 10 μm. **(D)** Average number of VGLUT-ir *punctae* on GnRH neurons in P10 *p140Cap* KO and WT mice. *n* = 3 WT mice; 3 *p140Cap* KO mice; at least 30 GnRH neurons were analyzed for each mouse. Unpaired, two-tailed *t*-test, *P* = 0.006. **(E)** Maximum intensity projections of z-stack images (20 serial image planes; z step size = 0.5 μm) of VGLUT-ir *punctae* (red) and GnRH neurons (green) in P60 *p140Cap* KO and WT mice. Arrows indicate VGLUT-ir *punctae* on GnRH neurons. Scale bar: 20 μm. **(F)** Average number of VGLUT-ir *punctae* on GnRH neurons in P60 *p140Cap* KO and WT mice. *n* = 3 WT mice; 3 *p140Cap* KO mice; at least 30 GnRH neurons were analyzed for each mouse. Unpaired, two-tailed *t*-test, *P* = 0.003. Data are represented as means ± SEM. * = *P* < 0.05, ** = *P* < 0.01, ns = not significant.

First, we examined the overall density of glutamatergic (VGLUT-ir) *punctae* in anatomically matched sections of the OVLT and POA by immunostaining on P60 *p140Cap* KO and WT animals. We found a significant reduction in their density in the absence of p140Cap (Figures 7A,B), indicating that p140Cap is required for efficient glutamatergic synaptogenesis in these areas. Then, we specifically determined the number of VGLUT-ir *punctae* in close apposition to the soma of GnRH neurons at P10 and P60 in *p140Cap* KO and WT mice. We observed a significant reduction in the average number of VGLUT-ir *punctae* per GnRH neuron at both P10 (Figures 7C,D) and P60 (Figures 7E,F) in *p140Cap* KO animals, as compared to WT. This result suggests that GnRH neurons of *p140Cap* KO animals are inefficiently innervated by glutamatergic synapses.

We also examined the density of GABAergic (VGAT-ir) *punctae* in close apposition to the perisomatic surface of GnRH neurons, comparing *p140Cap* KO and WT hypothalami, but we observed no significant difference at both P10 (Supplementary Figures 5A,B) and P60 (Supplementary Figures 5C,D). Considering that KP neurons represent the primary modulators of GnRH neurons, we finally assessed the number of VGLUT-ir and VGAT-ir *punctae* in close apposition to KP neurons in the RP3V of *p140Cap* KO and WT P120 female mice in the proestrus phase of the estrus cycle. We observed no difference in the number of both VGLUT-ir and VGAT-ir *punctae* comparing the two genotypes (Supplementary Figures 5E,F).

## Discussion

Here we report that the loss of the adaptor protein p140Cap is responsible for a striking reproductive defect in female mice, characterized by reduced number of litters, impaired ovulation, absent elevation of E2 and LH during proestrus, and delayed puberty onset. In the absence of p140Cap, adult hypothalamic GnRH neurons exhibit a mature morphology and normal responsiveness to KP stimulation, are reduced in number, project fewer axons to the ME, show reduced glutamatergic innervations, and fail to exert a proper gonadotropic action.

The failure to elevate E2 and LH during proestrus in *p140Cap* KO females implies that the loss of p140Cap impairs the regulation of the pre-ovulatory gonadotropin surge. This phenotype has a clear central hypothalamic origin as p140Cap is not expressed in the adult pituitary gland and ovaries, but it is expressed in various regions of the developing and adult brain, including the hypothalamus. Furthermore, the pituitary gland of *p140Cap* KO females is able to produce an LH surge upon administration of exogenous KP, and the ovaries of *p140Cap* KO mice retain their ability to undertake ovulation upon exogenous hormonal stimulation.

Focusing on the status of GnRH neurons at different ages, we observed that, in *p140Cap* KO adult females, the number of GnRH neurons and their projections to the ME are reduced, accompanied by a decreased expression of LH and GnRH mRNAs in the pituitary gland and hypothalamus, respectively. In contrast, at birth and early postnatal stages (P10), the number of GnRH neurons in *p140Cap* KO females is normal. Thus, the decline of GnRH neurons might occur between the juvenile and the adult stage. A similar loss of GnRH neurons, together with hypofertility or infertility, is a hallmark of a number of murine models of Kallmann syndrome and normosmotic idiopathic hypogonadotropic hypogonadism, such as homozygous Fgf8 and *Fgfr1* hypomorphs, in which GnRH neurons fail to emerge (Chung et al., 2008), *Sema3e* KO, in which a large fraction of GnRH neurons undergo apoptosis (Cariboni et al., 2015), *Prok2* KO, and *Prok2r* KO, in which GnRH neurons show defective migration (Matsumoto et al., 2006; Pitteloud et al., 2007). A loss of GnRH neurons was also observed in *Rabconnectin-3*α KO and heterozygous *Fgf8* hypomorphic mice (Zhang et al., 2015; Tata et al., 2017), although the cause of such loss in these models remains undetermined. Similarly, we were not able to identify the cause of the loss of GnRH neurons in *p140Cap* KO mice, as our data seem to exclude altered migration during embryonic development, impaired neurogenesis, and altered survival of GnRH neurons. However, we cannot rule out the possibility that, in the absence of p140Cap, GnRH neurons undergo apoptosis in a very specific and limited time window. Alternatively, it is possible that, in *p140Cap* KO mice, apoptosis of GnRH neurons occurs over an extended time window at a rate that is too low to be appreciated. Another hypothesis is that the loss of GnRH neurons in *p140Cap* KO mice is due to a defect in GnRH gene expression in a subpopulation of GnRH neurons. Importantly, the reduction in the number of total GnRH neurons is not accountable for the hypofertility phenotype observed in *p140Cap* KO female mice, as it has been shown that a ∼60% reduction in the number of total GnRH neurons is still compatible with normal puberty onset and fertility (Herbison et al., 2008; Zhang et al., 2015).

To clarify the mechanism by which p140Cap controls GnRH neuronal maturation and gonadotropic activity, we examined the expression of p140Cap at key time points of GnRH neuron development. At embryonic stages, a consistent fraction of migrating GnRH neurons express p140Cap, whereas in adult (P60) stages p140Cap expression is not observed in GnRH neurons but is evident in other hypothalamic neuronal populations of the OVLT and ARC. Nevertheless, at birth and in early postnatal life, the number and position of GnRH neurons show no significant changes in *p140Cap* KO mice, indicating that their ability to migrate and reach their final location is normal. At P10, GnRH neurons of *p140Cap* KO females show a delayed morphological maturation, with a larger fraction of them being multipolar, an index of delayed maturation (Cottrell et al., 2006; Ybarra et al., 2011; Tata et al., 2014). On the contrary, the adult GnRH neurons show a normal morphology, suggesting that the maturation delay has been overcome. An intriguing possibility is that the maturation delay and increased cell death/impaired *GnRH* gene expression of GnRH neurons observed in adult *p140Cap* KO mice concern only the subpopulation of GnRH neurons expressing p140Cap during embryonic development.

In *p140cap KO* mice, the adult GnRH neurons show normal immediate responsiveness to exogenous KP, suggesting that the hypofertility phenotype of *p140Cap* KO females does not depend on cell-autonomous defects of GnRH neurons. Furthermore, the number and fiber extension of KP neurons also appear normal in the absence of p140Cap. However, KP neurons function was not assessed. Thus, we cannot exclude that a misfunction of the KP system contributes to the observed gonadotropic phenotype in *p140Cap* KO females.

In the absence of p140Cap, we observe a lower density of VGLUT-ir *punctae* suggestive of reduced glutamatergic synapses in the OVLT and POA, and in particular on the GnRH neurons, at both P10 and P60. Thus, p140Cap may be required for the efficient formation and/or stabilization of glutamatergic synapses in these regions. We previously showed that p140Cap controls cytoskeleton dynamics at dendritic spines and participates in synaptic maturation and stability, based on acute knockdown and KO mouse models (Tomasoni et al., 2013; Repetto et al., 2014). This observation has been extended in recently published reports indicating that p140Cap acts pre- and post-synaptically to promote the formation and stabilization of glutamatergic synapses in the forebrain (Boyken et al., 2013; Li et al., 2017).

Several lines of evidence indicate that glutamatergic stimulation in the hypothalamus is critical for GnRH neurons activity and fertility: glutamate stimulates GnRH release from GT1-7 cells *in vitro* (Spergel et al., 1994; El-Etr et al., 2006) and from hypothalamic fragments *ex vivo* (Ondo et al., 1988)*; In vivo*, the number of VGLUT-ir punctae on GnRH neurons increases on proestrus, and blockage of glutamate neurotransmission on proestrus through glutamate receptor antagonists leads to a significant attenuation of the LH surge (Khan et al., 2010). However, it is currently unclear whether the GnRH release-promoting activity of glutamate depends on direct stimulation of GnRH neurons or the activation of dedicated circuits, that, in turn, promote and/or synchronize their activity. Such circuits include KP neurons of the RP3V and glutamatergic interconnected KNDY neurons (coexpressing KP, neurokinin B, and dynorphin A) of the ARC (Nagae et al., 2021; Nandankar et al., 2021). Some observations seem to exclude a direct role of glutamatergic stimulation on GnRH neurons: peripheral injection of NMDA fails to induce LH release in *Kiss1* KO mice (d’Anglemont de Tassigny et al., 2010) and mice lacking GluA2-containing AMPA receptors or all NMDA receptors only in GnRH neurons exhibit normal puberty onset and fertility (Shimshek et al., 2006). Nevertheless, in these mutant mice, other neurotransmitter receptors may have compensated for the lack of GluA2-containing AMPA receptors or NMDA receptors during development.

Given these considerations, the hypoactivity of the HPG axis in *p140Cap* KO female mice is probably due to non-cell-autonomous effects: either reduced glutamatergic stimulation of GnRH neurons or to a defective glutamatergic circuitry in the hypothalamus. Moreover, hypofertility of *p140Cap* KO females may be also due to a global maladaptation or secondary effects caused by the depletion of *p140Cap* in the hypothalamus.

We also found a normal density of VGAT-ir *punctae* in close apposition to GnRH neurons. This observation is in line with the recent recognition of the essential role of KP/GABA co-transmission onto GnRH neurons for ovulation (Piet et al., 2018) and suggests that, in mice, the action of glutamate on GnRH neurons is largely independent of the RP3V kisspeptidergic system. However, this does not exclude a possible important role of glutamate signaling at the level of the ARC KNDY neurons, which may have been impacted in the *p140Cap* KO thus indirectly affecting GnRH function. Notably, data from single-cell RNA-seq datasets indicate that at least a fraction of mouse ARC KNDY neurons express p140Cap (Campbell et al., 2017; Chen et al., 2017), and it has been shown that these neurons receive glutamatergic inputs which are likely to mediate the effects of E2 on these cells (Wang et al., 2018).

Synchronicity has been shown to be essential for efficient pulsatile secretion of GnRH, which, in turn, is required for an efficient gonadotropic function in females (Grachev and Goodman, 2016; Herbison, 2018). Interestingly, in the absence of p140Cap, altered synchronization of hippocampal neuron culture has been recently shown (Russo et al., 2019). Thus, the absence of p140Cap in the hypothalamus may affect both VGLUT-ir synaptic formation and the complex neuronal networks underlying GnRH neurons pulse generation and synchronization. Defining the exact effect of glutamatergic signaling in this network and specifically on GnRH neurons maturation and function will add relevant information to the mechanism underlying the control of GnRH secretion.

## Data Availability Statement

The raw data supporting the conclusions of this article will be made available by the authors, without undue reservation.

## Ethics Statement

The animal study was reviewed and approved by Italian Ministry of Health.

## Author Contributions

MC, IR, VZ, PG, AC, IF, ET, PD, and GRM conceived and designed the experiments. MC, IR, VZ, AA, SR, AM, IC, CA, RO, and FA performed the experiments. MC, IR, VZ, AA, SR, IC, CA, PG, RO, and FA analyzed the data. PG, AC, IF, ET, PD, and GRM contributed reagents, materials, and tools. MC, IR, VZ, ET, PD, and GRM wrote the manuscript. All authors contributed to the article and approved the submitted version.

## Conflict of Interest

The authors declare that the research was conducted in the absence of any commercial or financial relationships that could be construed as a potential conflict of interest.

## Publisher’s Note

All claims expressed in this article are solely those of the authors and do not necessarily represent those of their affiliated organizations, or those of the publisher, the editors and the reviewers. Any product that may be evaluated in this article, or claim that may be made by its manufacturer, is not guaranteed or endorsed by the publisher.

## Footnotes

1
http://imagej.nih.gov/ij/

## References

[B1] Beauvillain J. C. Tramu G. (1980). Immunocytochemical demonstration of LH-RH, somatostatin, and ACTH-like peptide in osmium-postfixed, resin-embedded median eminence. *J. Histochem. Cytochem.* 28 1014–1017. 10.1177/28.9.6157712 6157712

[B2] Boehm U. Bouloux P. M. Dattani M. T. De Roux N. Dodé C. Dunkel L. (2015). Expert consensus document: European Consensus Statement on congenital hypogonadotropic hypogonadism-pathogenesis, diagnosis and treatment. *Nat. Rev. Endocrinol.* 11 547–564. 10.1038/nrendo.2015.112 26194704

[B3] Boyken J. Grønborg M. Riedel D. Urlaub H. Jahn R. Chua J. J. E. (2013). Molecular profiling of synaptic vesicle docking sites reveals novel proteins but few differences between glutamatergic and GABAergic synapses. *Neuron* 78 285–297. 10.1016/j.neuron.2013.02.027 23622064

[B4] Brann D. W. Mahesh V. B. (1991). Endogenous excitatory amino acid involvement in the preovulatory and steroid-induced surge of gonadotropins in the female rat. *Endocrinology* 128 1541–1547. 10.1210/endo-128-3-1541 1900231

[B5] Caligioni C. S. (2009). Assessing reproductive status/stages in mice. *Curr. Protoc. Neurosci.* 48 1–6. 10.1002/0471142301.nsa04is48 19575469 PMC2755182

[B6] Campbell J. N. Macosko E. Z. Fenselau H. Pers T. H. Lyubetskaya A. Tenen D. (2017). A molecular census of arcuate hypothalamus and median eminence cell types. *Nat. Neurosci.* 20 484–496. 10.1038/NN.4495 28166221 PMC5323293

[B7] Cariboni A. André V. Chauvet S. Cassatella D. Davidson K. Caramello A. (2015). Dysfunctional SEMA3E signaling underlies gonadotropin-releasing hormone neuron deficiency in Kallmann syndrome. *J. Clin. Invest.* 125 2413–2428. 10.1172/JCI78448 25985275 PMC4497752

[B8] Cariboni A. Maggi R. (2006). Kallmann’s syndrome, a neuronal migration defect. *Cell. Mol. Life Sci.* 63 2512–2526. 10.1007/s00018-005-5604-3 16952059 PMC11136178

[B9] Cariboni A. Maggi R. Parnavelas J. G. (2007). From nose to fertility: the long migratory journey of gonadotropin-releasing hormone neurons. *Trends Neurosci.* 30 638–644. 10.1016/j.tins.2007.09.002 17981344

[B10] Chen R. Wu X. Jiang L. Zhang Y. (2017). Single-Cell RNA-seq reveals hypothalamic cell diversity. *Cell Rep.* 18 3227–3241. 10.1016/J.CELREP.2017.03.004 28355573 PMC5782816

[B11] Chan Y. M. Lippincott M. F. Butler J. P. Sidhoum V. F. Li C. X. Plummer L. (2014). Exogenous kisspeptin administration as a probe of GnRH neuronal function in patients with idiopathic hypogonadotropic hypogonadism. *J. Clin. Endocrinol. Metab.* 99, E2762–E2771. 10.1210/jc.2014-2233 25226293 PMC4255107

[B12] Chin L. S. Nugent R. D. Raynor M. C. Vavalle J. P. Li L. (2000). SNIP, a novel SNAP-25-interacting protein implicated in regulated exocytosis. *J. Biol. Chem.* 275 1191–1200. 10.1074/jbc.275.2.1191 10625663

[B13] Chung W. C. Moyle S. S. Tsai P. S. (2008). Fibroblast growth factor 8 signaling through fibroblast growth factor receptor 1 is required for the emergence of gonadotropin-releasing hormone neurons. *Endocrinology* 149 4997–5003. 10.1210/EN.2007-1634 18566132 PMC2582917

[B14] Clarkson J. d’Anglemont de Tassigny X. Colledge W. H. Caraty A. Herbison A. E. (2009). Distribution of kisspeptin neurones in the adult female mouse brain. *J. Neuroendocrinol.* 21 673–682. 10.1111/j.1365-2826.2009.01892.x 19515163

[B15] Clarkson J. Herbison A. E. (2006). Development of GABA and glutamate signaling at the GnRH neuron in relation to puberty. *Mol. Cell. Endocrinol.* 254–255 32–38. 10.1016/j.mce.2006.04.036 16781054

[B16] Cottrell E. C. Campbell R. E. Han S. K. Herbison A. E. (2006). Postnatal remodeling of dendritic structure and spine density in gonadotropin-releasing hormone neurons. *Endocrinology* 147 3652–3661. 10.1210/en.2006-0296 16644918

[B17] d’Anglemont de Tassigny X. Ackroyd K. J. Chatzidaki E. E. Colledge W. H. (2010). Kisspeptin signaling is required for peripheral but not central stimulation of gonadotropin-releasing hormone neurons by NMDA. *J. Neurosci.* 30 8581–8590. 10.1523/JNEUROSCI.5486-09.2010 20573904 PMC6634616

[B18] Di Stefano P. Damiano L. Cabodi S. Aramu S. Tordella L. Praduroux A. (2007). p140Cap protein suppresses tumour cell properties, regulating Csk and Src kinase activity. *EMBO J.* 26 2843–2855. 10.1038/sj.emboj.7601724 17525734 PMC1894765

[B19] El-Etr M. Akwa Y. Baulieu E. E. Schumacher M. (2006). The neuroactive steroid pregnenolone sulfate stimulates the release of gonadotropin-releasing hormone from GT1-7 hypothalamic neurons, through N-methyl-D-aspartate receptors. *Endocrinology* 147 2737–2743. 10.1210/en.2005-1191 16513833

[B20] Forni P. E. Taylor-Burds C. Melvin V. S. Williams T. Wray S. (2011). Neural crest and ectodermal cells intermix in the nasal placode to give rise to GnRH-1 neurons, sensory neurons, and olfactory ensheathing cells. *J. Neurosci.* 31 6915–6927. 10.1523/JNEUROSCI.6087-10.2011 21543621 PMC3101109

[B21] Franceschini I. Yeo S. H. Beltramo M. Desroziers E. Okamura H. Herbison A. E. (2013). Immunohistochemical evidence for the presence of various kisspeptin isoforms in the mammalian brain. *J. Neuroendocrinol.* 25 839–851. 10.1111/jne.12069 23822722

[B22] Giacobini P. Messina A. Morello F. Ferraris N. Corso S. Penachioni J. (2008). Semaphorin 4D regulates gonadotropin hormonereleasing hormone-1 neuronal migration through PlexinBl-Met complex. *J. Cell Biol.* 183 555–566. 10.1083/jcb.200806160 18981235 PMC2575794

[B23] Grachev P. Goodman R. L. (2016). The GnRH Pulse Generator. *AIMScMed. Sci.* 3 359–385. 10.3934/medsci.2016.4.359

[B24] Grasso S. Chapelle J. Salemme V. Aramu S. Russo I. Vitale N. (2018). Erratum: author Correction: the scaffold protein p140Cap limits ERBB2-mediated breast cancer progression interfering with Rac GTPase-controlled circuitries (Nature communications (2017) 8 (14797)). *Nat. Commun.* 9:16203. 10.1038/ncomms16203 29600801 PMC5882465

[B25] Harter C. J. L. Kavanagh G. S. Smith J. T. (2018). The role of kisspeptin neurons in reproduction and metabolism. *J. Endocrinol.* 238 R173–R183. 10.1530/JOE-18-0108 30042117

[B26] Herbison A. E. (2016). Control of puberty onset and fertility by gonadotropin-releasing hormone neurons. *Nat. Rev. Endocrinol.* 12 452–466. 10.1038/nrendo.2016.70 27199290

[B27] Herbison A. E. (2018). The gonadotropin-releasing hormone pulse generator. *Endocrinology* 159 3723–3736. 10.1210/en.2018-00653 30272161

[B28] Herbison A. E. Porteous R. Pape J. R. Mora J. M. Hurst P. R. (2008). Gonadotropin-releasing hormone neuron requirements for puberty, ovulation, and fertility. *Endocrinology* 149 597–604. 10.1210/en.2007-1139 18006629 PMC6101186

[B29] Iremonger K. J. Constantin S. Liu X. Herbison A. E. (2010). Glutamate regulation of GnRH neuron excitability. *Brain Res.* 1364 35–43. 10.1016/j.brainres.2010.08.071 20807514

[B30] Ito H. Atsuzawa K. Sudo K. Di Stefano P. Iwamoto I. Morishita R. (2008). Characterization of a multidomain adaptor protein, p140Cap, as part of a pre-synaptic complex. *J. Neurochem.* 107 61–72. 10.1111/j.1471-4159.2008.05585.x 18662323

[B31] Jasoni C. L. Porteous R. W. Herbison A. E. (2009). Anatomical location of mature GnRH neurons corresponds with their birthdate in the developing mouse. *Dev. Dyn.* 238 524–531. 10.1002/DVDY.21869 19191221

[B32] Jaworski J. Kapitein L. C. Gouveia S. M. Dortland B. R. Wulf P. S. Grigoriev I. (2009). Dynamic microtubules regulate dendritic spine morphology and synaptic plasticity. *Neuron* 61 85–100. 10.1016/j.neuron.2008.11.013 19146815

[B33] Kennedy T. G. Armstrong D. T. (1973). Lack of specificity for the extra-ovarian prolactin effect on vaginal mucification in rats. *Endocrinology* 92 847–852. 10.1210/endo-92-3-847 4349653

[B34] Khan M. de Sevilla L. Mahesh V. B. Brann D. W. (2010). Enhanced glutamatergic and decreased gabaergic synaptic appositions to GnRH neurons on proestrus in the rat: modulatory effect of aging. *PLoS One* 5:e10172. 10.1371/journal.pone.0010172 20418960 PMC2854717

[B35] León S. Barroso A. Vázquez M. J. García-Galiano D. Manfredi-Lozano M. Ruiz-Pino F. (2016). Direct actions of kisspeptins on GnRH neurons permit attainment of fertility but are insufficient to fully preserve gonadotropic axis activity. *Sci. Rep.* 6:19206. 10.1038/srep19206 26755241 PMC4709743

[B36] Li M. Y. Miao W. Y. Wu Q. Z. He S. J. Yan G. Yang Y. (2017). A critical role of presynaptic cadherin/catenin/p140Cap complexes in stabilizing spines and functional synapses in the neocortex. *Neuron* 94 1155–1172.e8. 10.1016/j.neuron.2017.05.022 28641114

[B37] Macchi C. Steffani L. Oleari R. Lettieri A. Valenti L. Dongiovanni P. (2017). Iron overload induces hypogonadism in male mice via extrahypothalamic mechanisms. *Mol. Cell. Endocrinol.* 454, 135–145. 10.1016/J.MCE.2017.06.019 28648620

[B38] Matsumoto S. I. Yamazaki C. Masumoto K. H. Nagano M. Naito M. Soga T. (2006). Abnormal development of the olfactory bulb and reproductive system in mice lacking prokineticin receptor PKR2. *Proc. Natl. Acad. Sci. U. S. A.* 103 4140–4145. 10.1073/PNAS.0508881103 16537498 PMC1449660

[B39] Nagae M. Uenoyama Y. Okamoto S. Tsuchida H. Ikegami K. Goto T. (2021). Direct evidence that KNDy neurons maintain gonadotropin pulses and folliculogenesis as the GnRH pulse generator. *Proc. Natl. Acad. Sci. U. S. A.* 118:e2009156118. 10.1073/pnas.2009156118 33500349 PMC7865162

[B40] Nandankar N. Negron A. L. Wolfe A. Levine J. E. Radovick S. (2021). Deficiency of arcuate nucleus kisspeptin results in post-pubertal central hypogonadism. *Am. J. Physiol. Endocrinol. Metab.* 321 E264–E280. 10.1152/ajpendo.00088.2021 34181485 PMC8410100

[B41] Naulé L. Picot M. Martini M. Parmentier C. Hardin-Pouzet H. Keller M. (2014). Neuroendocrine and behavioral effects of maternal exposure to oral bisphenol A in female mice. *J. Endocrinol.* 220 375–388. 10.1530/JOE-13-0607 24403293

[B42] Oleari R. André V. Lettieri A. Tahir S. Roth L. Paganoni A. (2021). A novel SEMA3G mutation in two siblings affected by syndromic GnRH deficiency. *Neuroendocrinology* 111 421–441. 10.1159/000508375 32365351

[B43] Ondo J. G. Wheeler D. D. Dom R. M. (1988). Hypothalamic site of action for N-methyl-D-aspartate (n.d.) on LH secretion. *Life Sci.* 43 2283–2286. 10.1016/0024-3205(88)90422-5 3062296

[B44] Paxinos G. Franklin K. B. J. (2001). *The Mouse Brain in Stereotaxic Coordinates*, 2nd Edn. Cambridge: Academic Press. 10.1016/s0969-9961(08)00084-3

[B45] Piet R. Kalil B. McLennan T. Porteous R. Czieselsky K. Herbison A. E. (2018). Dominant neuropeptide cotransmission in kisspeptin-GABA regulation of GnRH neuron firing driving ovulation. *J. Neurosci.* 38 6310–6322. 10.1523/JNEUROSCI.0658-18.2018 29899026 PMC6596098

[B46] Pitteloud N. Zhang C. Pignatelli D. Li J. D. Raivio T. Cole L. W. (2007). Loss-of-function mutation in the prokineticin 2 gene causes Kallmann syndrome and normosmic idiopathic hypogonadotropic hypogonadism. *Proc. Natl. Acad. Sci. U. S. A.* 104 17447–17452. 10.1073/PNAS.0707173104 17959774 PMC2077276

[B47] Repetto D. Camera P. Melani R. Morello N. Russo I. Calcagno E. (2014). p140Cap regulates memory and synaptic plasticity through src-mediated and citron-N-mediated actin reorganization. *J. Neurosci.* 34 1542–1553. 10.1523/JNEUROSCI.2341-13.2014 24453341 PMC6705312

[B48] Rodriguez I. Araki K. Khatib K. Martinou J. C. Vassalli P. (1997). Mouse vaginal opening is an apoptosis-dependent process which can be prevented by the overexpression of Bcl2. *Dev. Biol.* 184 115–121. 10.1006/dbio.1997.8522 9142988

[B49] Russo I. Gavello D. Menna E. Vandael D. Veglia C. Morello N. (2019). P140CAP regulates GABAergic synaptogenesis and development of hippocampal inhibitory circuits. *Cereb. Cortex* 29 91–105. 10.1093/cercor/bhx306 29161354

[B50] Shimshek D. R. Bus T. Grinevich V. Single F. N. Mack V. Sprengel R. (2006). Impaired reproductive behavior by lack of GluR-B containing AMPA receptors but not of NMDA receptors in hypothalamic and septal neurons. *Mol. Endocrinol.* 20 219–231. 10.1210/me.2005-0262 16099814

[B51] Skrapits K. Kanti V. Savanyú Z. Maurnyi C. Szenci O. Horváth A. (2015). Lateral hypothalamic orexin and melanin-concentrating hormone neurons provide direct input to gonadotropin-releasing hormone neurons in the human. *Front. Cell. Neurosci.* 9:348. 10.3389/FNCEL.2015.00348 26388735 PMC4559643

[B52] Smyth C. Wilkinson M. (1994). A critical period for glutamate receptor-mediated induction of precocious puberty in female rats. *J. Neuroendocrinol.* 6 275–284. 10.1111/j.1365-2826.1994.tb00583.x 7920593

[B53] Spergel D. J. (2019a). Modulation of gonadotropin-releasing hormone neuron activity and secretion in mice by non-peptide neurotransmitters, gasotransmitters, and gliotransmitters. *Front. Endocrinol.* 10:329. 10.3389/fendo.2019.00329 31178828 PMC6538683

[B54] Spergel D. J. (2019b). Neuropeptidergic modulation of GnRH neuronal activity and GnRH secretion controlling reproduction: insights from recent mouse studies. *Cell Tissue Res.* 375 179–191. 10.1007/s00441-018-2893-z 30078104

[B55] Spergel D. J. Krsmanovic L. Z. Stojilkovic S. S. Catt K. J. (1994). Glutamate modulates [Ca2+]i and gonadotropin-releasing hormone secretion in immortalized hypothalamic GT1-7 neurons. *Neuroendocrinology* 59 309–317. 10.1159/000126672 7911229

[B56] Stefano P. di Cabodi S. Erba E. B. Margaria V. Bergatto E. Giuffrida M. G. (2004). p130Cas-associated protein (p140Cap) as a new tyrosine-phosphorylated protein involved in cell spreading. *Mol. Biol. Cell* 15, 787–800. 10.1091/mbc.E03-09-0689 14657239 PMC329393

[B57] Tata B. Huijbregts L. Jacquier S. Csaba Z. Genin E. Meyer V. (2014). Haploinsufficiency of Dmxl2, encoding a synaptic protein, causes infertility associated with a loss of GnRH neurons in mouse. *PLoS Biol.* 12:e1001952. 10.1371/journal.pbio.1001952 25248098 PMC4172557

[B58] Tata B. K. Harbulot C. Csaba Z. Peineau S. Jacquier S. De Roux N. (2017). Rabconnectin-3α is required for the morphological maturation of GnRH neurons and kisspeptin responsiveness. *Sci. Rep.* 7:42463. 10.1038/srep42463 28209974 PMC5314327

[B59] Tomasoni R. Repetto D. Morini R. Elia C. Gardoni F. Di Luca M. (2013). SNAP-25 regulates spine formation through postsynaptic binding to p140Cap. *Nat. Commun.* 4:2136. 10.1038/ncomms3136 23868368

[B60] Urbanski H. F. (1990). A role for N-methyl-D-aspartate (n.d.) receptors in the control of LH secretion and initiation of female puberty. *Endocrinology* 126 1774–1776. 10.1210/endo-126-3-1774 1968384

[B61] Wang L. Burger L. L. Greenwald-Yarnell M. L. Myers M. G. Moenter S. M. (2018). Glutamatergic transmission to hypothalamic kisspeptin neurons is differentially regulated by estradiol through estrogen receptor α in adult female mice. *J. Neurosci.* 38, 1061–1072. 10.1523/JNEUROSCI.2428-17.2017 29114074 PMC5792470

[B62] Whittaker D. E. Oleari R. Gregory L. C. le Quesne-Stabej P. Williams H. J. Torpiano J. G. (2021). A recessive PRDM13 mutation results in congenital hypogonadotropic hypogonadism and cerebellar hypoplasia. *J. Clin. Invest.* 131:e141587. 10.1172/JCI141587 34730112 PMC8670848

[B63] Ybarra N. Hemond P. J. O’Boyle M. P. Suter K. J. (2011). Spatially selective, testosterone-independent remodeling of dendrites in gonadotropin-releasing hormone (GnRH) neurons prepubertally in male rats. *Endocrinology* 152 2011–2019. 10.1210/en.2010-0871 21343259 PMC3075933

[B64] Zhang W. Johnson J. I. Tsai P.-S. (2015). Fgf8-deficient mice compensate for reduced GnRH neuronal population and exhibit normal testicular function. *Front. Endocrinol.* 6:22. 10.3389/fendo.2015.00151 26441841 PMC4585285

