# Supplementary material for: p140Cap Controls Female Fertility in Mice Acting via Glutamatergic Afference on Hypothalamic Gonadotropin-Releasing Hormone Neurons
Source: Front Neurosci. 2022 Feb 14;16:744693. doi: 10.3389/fnins.2022.744693 (PMC8884249; doi:10.3389/fnins.2022.744693)
Supplement: Supplementary file 1 [file Image_1.pdf]

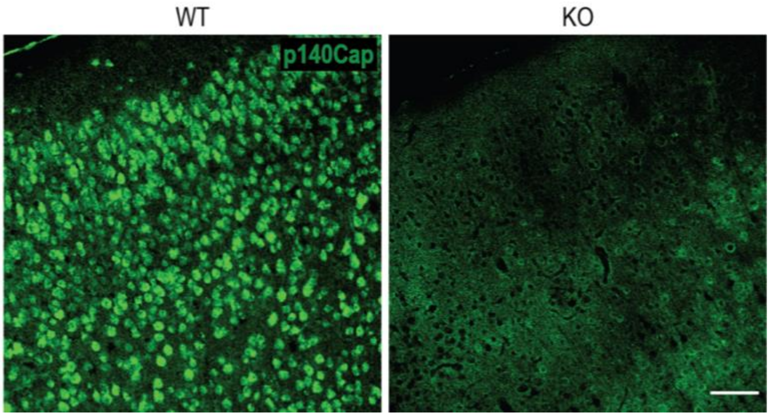

Supplementary Figure 1. Mouse anti-p140Cap antibody specificity.

Maximum intensity projections of z-stack images (5 serial image planes; z step size = 1 µm) of p140Cap in the cortex of *p140Cap* KO and WT P120 mice. Scale bar: 100 µm.

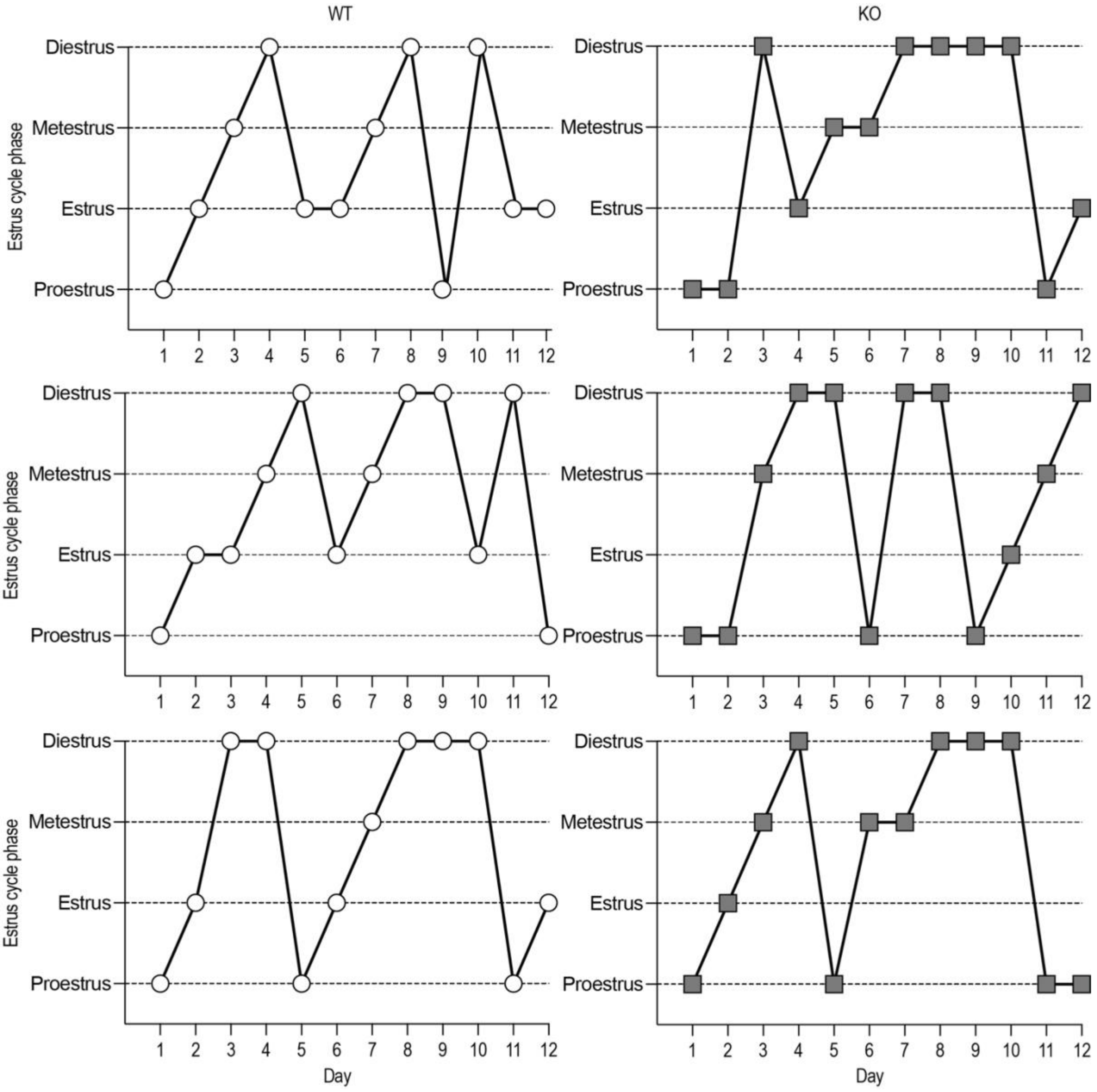

Supplementary Figure 2. ***p140Cap*** **KO females display abnormal estrous cyclicity.**

Representative estrous cycle transitions in *p140Cap* KO and WT adult mice. Each graph shows the transitions observed in a single animal over 12 days. The phase of the estrus cycle was determined as described in the material and methods section.

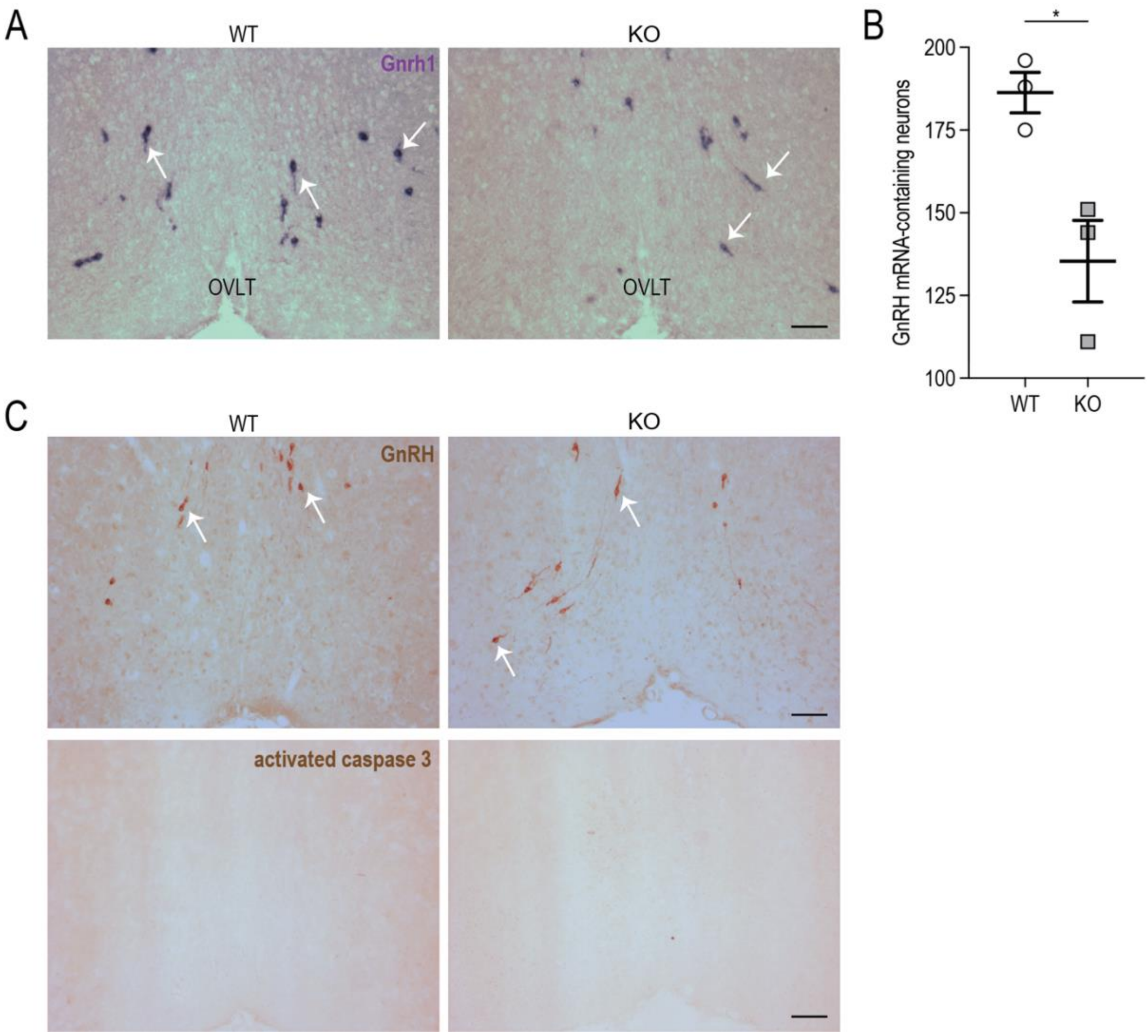

Supplementary Figure 3. ***p140Cap*** **KO female mice show a reduced number of Gnrh1 mRNA-containing neurons at P60 and unaltered cell death in the MPOA at P30.**

(A) *In situ* hybridization for the *Gnrh1* transcript in the MPOA of *p140Cap* KO and WT P60 female mice. Arrows indicate *Gnrh1*-expressing neurons. Scale bar: 250 µm. (B) Number of Gnrh1-expressing neurons in *p140Cap* KO and WT P60 female mice. n = 3 WT mice; 3 *p140Cap* KO mice. Unpaired, two-tailed t test, P = 0.02. (C) Immunostaining of GnRH neurons (upper panels) and apoptotic cells (lower panels) in adjacent sections of the MPOA of *p140Cap* KO and WT P30 female mice. Arrows indicate *GnRH*-ir neurons. Scale bar: 250 µm. OVLT = organum vasculosum of lamina terminalis. Data are represented as means±SEM. * = P<0.05, ** = P<0.01, *** = P<0.001, ns = not significant.

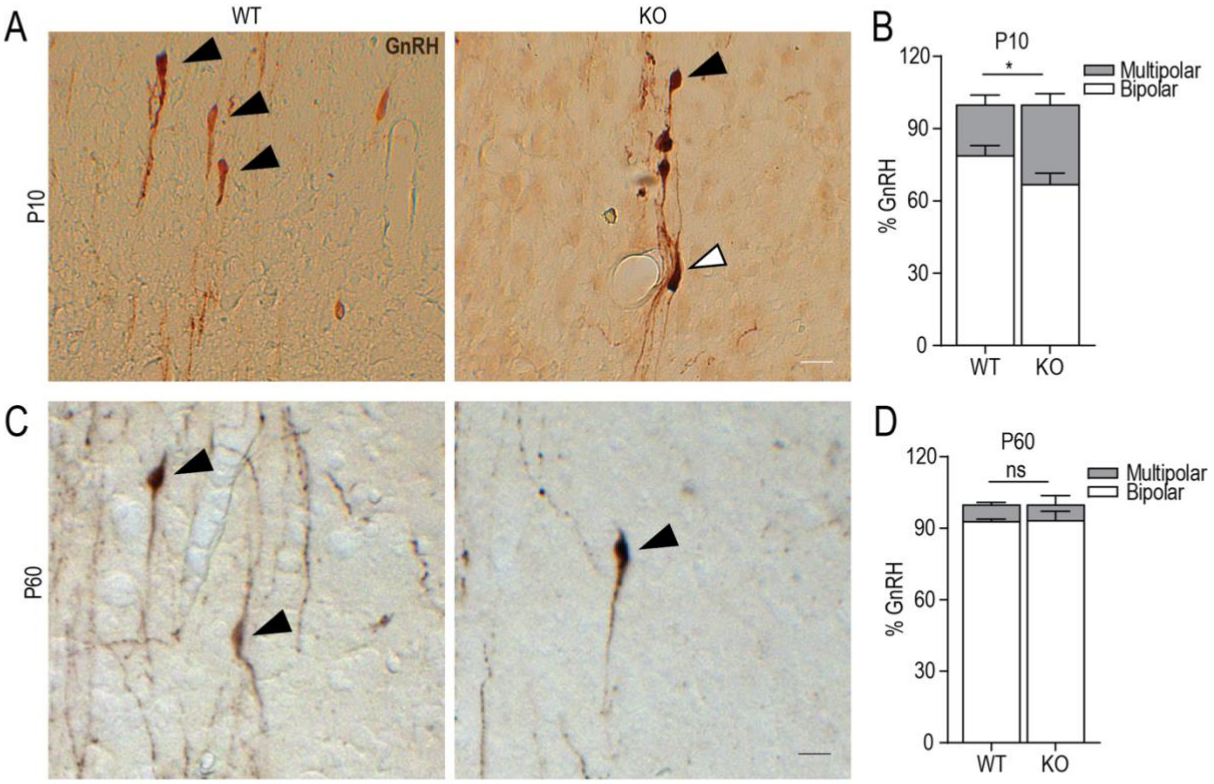

Supplementary Figure 4. GnRH neurons in *p140Cap* KO mice show delayed morphological maturation.

(A) Immunostaining of GnRH neurons in *p140Cap* KO and WT P10 mice. Scale bar: 20 µm. (B) Fraction of unipolar/bipolar (1 or 2 visible extensions) and multipolar (more than 2 visible extensions) GnRH neurons in *p140Cap* KO and WT P10 mice. n = 125 WT neurons; 180 *p140Cap* KO neurons. Unpaired, two-tailed t test, P = 0.05 (C) Immunostaining of GnRH neurons in *p140Cap* KO and WT P60 mice. Scale bar: 20 µm. (D) Fraction of bipolar and multipolar GnRH neurons in *p140Cap* KO and WT P60 mice. n = 130 WT neurons; 90 *p140Cap* KO neurons. Unpaired, two-tailed t test, P = 0.29. Black arrowheads indicate bipolar GnRH neurons, white arrowheads indicate multipolar GnRH neurons. Scale bars: 20 µm. Data are represented as means±SEM. * = P<0.05, ** = P<0.01, *** = P<0.001, ns = not significant.

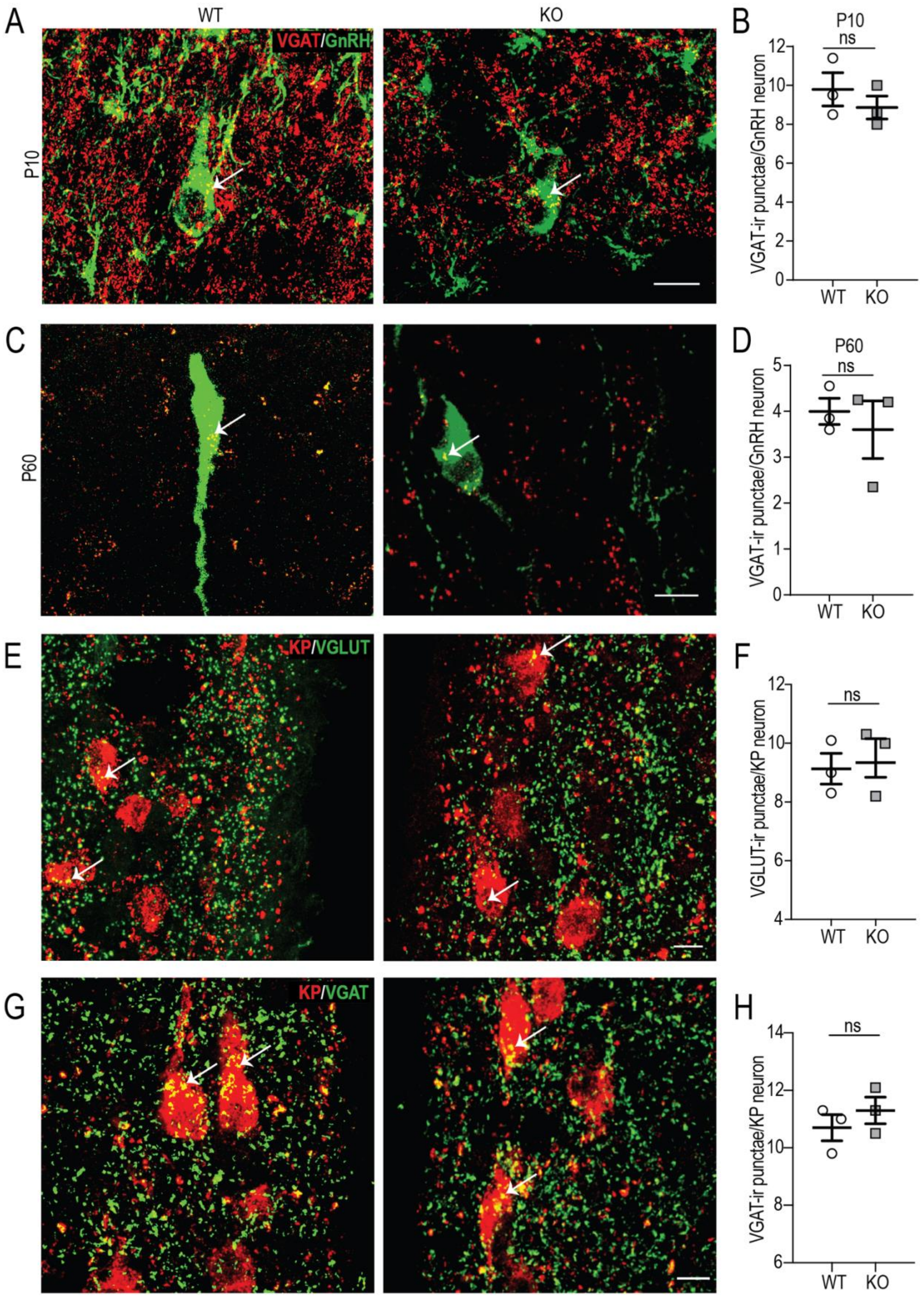

Supplementary Figure 5. *p140Cap* KO mice show normal GABAergic *punctae* on GnRH neurons and normal glutamatergic and GABAergic punctae on KP neurons.

(A) Maximum intensity projections of z-stack images (20 serial image planes; z step size = 0.5 μm) of VGAT-ir *punctae* (red) and GnRH neurons (green) in *p140Cap* KO and WT P10 mice. Arrows indicate VGAT-ir *punctae* on GnRH neurons. Scale bar: 10 μm. (B) Average number of VGAT-ir *punctae* on GnRH neurons in *p140Cap* KO and WT P10 mice. n = 3 WT mice; 3 *p140Cap* KO mice; at least 20 GnRH neurons

were analyzed for each mouse. Unpaired, two-tailed t test, P = 0.42. (C) Maximum intensity projections of z-stack images (20 serial image planes; z step size = 0.5 µm) of VGAT-ir *punctae* (red) and GnRH neurons (green) in *p140Cap* KO and WT P60 mice. Arrows indicate VGABA-ir *punctae* on GnRH neurons. Scale bar: 10 µm. (D) Average number of VGAT-ir *punctae* on GnRH neurons in *p140Cap* KO and WT P60 mice. n = 3 WT mice; 3 *p140Cap* KO mice; at least 20 GnRH neurons were analyzed for each mouse. Mann-Whitney test, P > 0.99. (E) Maximum intensity projections of z-stack images (10 serial image planes; z step size = 0.5 µm) of VGLUT-ir *punctae* (green) and KP neurons (red) in P60 *p140Cap* KO and WT mice. Arrows indicate VGLUT-ir *punctae* on KP neurons. Scale bar: 10 µm. (F) Average number of VGAT-ir *punctae* on KP neurons in *p140Cap* KO and WT P60 mice. n = 3 WT mice; 3 *p140Cap* KO mice; at least 50 GnRH neurons were analyzed for each mouse. Unpaired, two-tailed t test, P = 0.56. (G) Maximum intensity projections of z-stack images (10 serial image planes; z step size = 0.5 µm) of VGAT-ir *punctae* (green) and KP neurons (red) in *p140Cap* KO and WT P60 mice. Arrows indicate VGAT-ir *punctae* on KP neurons. Scale bar: 10 µm. (B) Average number of VGAT-ir *punctae* on KP neurons in *p140Cap* KO and WT P60 mice. n = 3 WT mice; 3 *p140Cap* KO mice; at least 50 GnRH neurons were analyzed for each mouse. Unpaired, two-tailed t test, P = 0.34. Data are represented as means±SEM. * = P<0.05, ** = P<0.01, *** = P<0.001, ns = not significant.
